# Whole-brain analytic measures of network communication reveal increased structure-function correlation in right temporal lobe epilepsy

**DOI:** 10.1016/j.nicl.2016.05.010

**Published:** 2016-05-19

**Authors:** Jonathan Wirsich, Alistair Perry, Ben Ridley, Timothée Proix, Mathieu Golos, Christian Bénar, Jean-Philippe Ranjeva, Fabrice Bartolomei, Michael Breakspear, Viktor Jirsa, Maxime Guye

**Affiliations:** a Aix-Marseille Université, CNRS, CRMBM UMR 7339, 13385 Marseille, France; b APHM, Hôpitaux de la Timone, Pôle d'imagerie Médicale, CEMEREM, 13005 Marseille, France; c Aix-Marseille Université, Institut de Neurosciences des Systèmes, 13385 Marseille, France; d INSERM, UMR_S 1106, 13385 Marseille, France; e APHM, Hôpitaux de la Timone, Pôle de Neurosciences Cliniques, Service de Neurophysiologie Clinique, 13005 Marseille, France; f Centre for Healthy Brain Ageing (CHeBA), School of Psychiatry, University of New South Wales, Sydney, NSW, Australia; g School of Psychiatry, University of New South Wales, Sydney, NSW 2052, Australia; h Systems Neuroscience Group, QIMR Berghofer Medical Research Institute, 300 Herston Road, Herston, QLD 4006, Australia; i Metro North Mental Health Services, Brisbane, QLD 4006, Australia

**Keywords:** CSD, constrained spherical deconvolution, CSF, cerebrospinal fluid, dMRI, diffusion magnetic resonance imaging, FA, fractional anisotropy, FCA, analytic functional connectivity, FCD, functional connectivity dynamics, FOD, fiber orientation distribution, NBS, network based statistics, rsfMRI, resting state functional magnetic resonance imaging, rTLE, right temporal lobe epilepsy, Structural connectivity, Functional connectivity, Temporal lobe epilepsy, Network communication, Rich club, Network based statistics

## Abstract

The in vivo structure-function relationship is key to understanding brain network reorganization due to pathologies. This relationship is likely to be particularly complex in brain network diseases such as temporal lobe epilepsy, in which disturbed large-scale systems are involved in both transient electrical events and long-lasting functional and structural impairments. Herein, we estimated this relationship by analyzing the correlation between structural connectivity and functional connectivity in terms of analytical network communication parameters. As such, we targeted the gradual topological structure-function reorganization caused by the pathology not only at the whole brain scale but also both in core and peripheral regions of the brain.

We acquired diffusion (dMRI) and resting-state fMRI (rsfMRI) data in seven righ*t*-lateralized TLE (rTLE) patients and fourteen healthy controls and analyzed the structure-function relationship by using analytical network communication metrics derived from the structural connectome.

In rTLE patients, we found a widespread hypercorrelated functional network. Network communication analysis revealed greater unspecific branching of the shortest path (search information) in the structural connectome and a higher global correlation between the structural and functional connectivity for the patient group. We also found evidence for a preserved structural rich-club in the patient group. In sum, global augmentation of structure-function correlation might be linked to a smaller functional repertoire in rTLE patients, while sparing the central core of the brain which may represent a pathway that facilitates the spread of seizures.

## Highlights

• rTLE patients exhibit increased mean search information compared controls.
• Structural search information best predicts functional connectivity in both groups.
• Whole brain structure-function correlation is increased in rTLE patients.
• Structure-function correlation differs in brain periphery but not in the rich club.

## 1 Introduction

Characterization of the structure-function relationship in brain networks is crucial to the understanding of both normal and diseased brain organization. This relationship can be defined by correlating functional and structural connectivity (Honey et al., 2009). The complete set of structural or functional links between brain areas has been referred to as the structural or functional connectome (Sporns et al., 2005) and can be analyzed by graph theoretical measures (Achard et al., 2006, Sporns, 2013). Graph theory holds promise as a method to reveal pathological reorganization of brain networks (Fornito et al., 2015, Guye et al., 2010). When the network is altered in pathologies, localized structural changes in the brain are likely to result in complex direct and indirect functional alterations at various different scales and levels of integration.

This propagation of local changes through the network renders the structure-function relationship highly non-trivial. Here we study the network changes in temporal lobe epilepsy (TLE), whilst taking into account the complexity of indirect structure-function correlations (Damoiseaux and Greicius, 2009) by means of a graph analytic network communication approach (Goñi et al., 2014). TLE is now regarded as a pathology involving areas not only participating in a localized epileptogenic network (producing transient electrical abnormalities) but also having long-lasting large scale network effects over the whole brain (Bartolomei et al., 2001, Laufs, 2012, Spencer, 2002). These effects have been revealed and quantified mostly by in vivo MRI studies, accessing both the structural and the functional organization of the whole brain (Bernhardt et al., 2013).

Structural alterations of a priori brain subnetworks have been assessed in epileptic patients using graph analysis, finding widespread subcortical alterations in epileptic subnetworks (Bonilha et al., 2012). In contrast, a more global approach comparing whole brain structural connectivity between patients with bilateral TLE and controls identified a network of structural connectivity alterations in the temporal lobe distributed to different networks for left and right TLE (Besson et al., 2014). Substrates of these structural alterations are still debated, though studies employing post mortem histology in TLE show the presence of white matter damage, neuronal loss and gliosis both within (Thom et al., 2000, Thom et al., 2001) and beyond epileptogenic regions (Blanc et al., 2011), most likely caused by the propagation of recurrent epileptic seizures throughout the brain.

From a functional perspective, rsfMRI studies in epileptic patients reported various, widespread and complex patterns of increased and decreased functional connectivity, effecting key features of large scale networks such as the default mode network (Haneef et al., 2012, Liao et al., 2010, Pittau et al., 2012, Voets et al., 2012). Discrepancies in reported functional brain reorganization could be related to the broad range of methodological approaches used in these studies (e.g. whole brain network corrected analysis vs. a-priori ROI analysis), which make it difficult to reach a synthetic interpretation of these results (Centeno et al., 2014, Constable et al., 2013).

In epilepsy, modeling the brain as a graph can be of use in assessing the link between structural and functional connectivity (Bernhardt et al., 2013, Guye et al., 2008). Several recent studies have endeavored to combine structural and functional approaches in epilepsy (Chiang et al., 2015, Douw et al., 2015, Vaessen et al., 2014, Voets et al., 2012, Zhang et al., 2011). Function-structure relationship has been studied in the epileptic brain in generalized epilepsies (Zhang et al., 2011), frontal lobe epilepsy (Vaessen et al., 2014) and in TLE (Voets et al., 2012). While focusing only on limbic and pre-limbic structures Chiang et al. (2015) found decreased structural-functional relationship in TLE. Douw et al. (2015) observed higher anatomical between-module connectivity which correlated with default mode disintegration in TLE compared to a control group.

Structural parameters of brain networks such as Euclidian distance or the combination of more complex graph analytical metrics of network communication such as path length, search information and path transitivity have also provided good predictions of functional connectivity in the normal brain (Goñi et al., 2014). Considering a structural link in the context of its neighbors makes structure-function analysis more sensitive to accumulated small structural changes which trigger widespread functional connectivity changes across the network. As such, we hypothesize that these metrics could help to better characterize structural-functional reorganization in TLE, particularly in the context of epilepsy where dMRI tractography cannot grasp the full spectrum of structural change induced by the pathology. For example, gradual linear structural changes (secondary effects of both ictal and interictal epileptic phenomena) such as demyelination may not be visible to probabilistic tractography, but by their distributed effect on functional connectivity they may be discernable via changes in structure-function relationship. The role of highly connected regions has also been stressed out recently. Indeed meta-analysis of graph theoretical studies has revealed that most of pathological lesions in the brain are linked to ‘hubs’ (Crossley et al., 2014). In the same line, recently, a “rich club” architecture has been proposed to describe the highly inter-connected core of hubs inside the brain (van den Heuvel and Sporns, 2011), which was found to be modified by pathology (Fornito et al., 2015, van den Heuvel et al., 2013). This structural core overlaps with propagation networks of epileptic activity in TLE, for example the precuneus (Arthuis et al., 2009) and as such might undergo structural and functional alterations in TLE patients, though this has yet to be demonstrated.

To our knowledge there is no study that directly evaluates the edgewise structure-function relationship at the whole brain scale, as well as within the rich-club and pathologically altered functional sub-networks in patients while taking into account network-based communication processes. In this context, we aimed to determine structure-function relationship within altered brain networks observed in rTLE. To do so we used a framework of several analytic approaches at different scales in a group of rTLE patients compared to a group of age and sex matched controls. First, structural and functional connectivity derived from dMRI/rs-fMRI data were used to examine whole brain reorganization in rTLE by using the Network Based Statistics (NBS) approach (Zalesky et al., 2010a), and by extracting the rich club structure of the brain. Second, to characterize whole brain functional-structural relationship in altered brains of rTLE patients, we sought to predict the functional connectivity from i) graph-analytical edgewise metrics derived from structural data (Goñi et al., 2014), and ii) Euclidian distance, and by comparing these results against current analytical and simulation models (Robinson et al., 2014). Third, we aimed to analyze structural-functional relationship within the altered functional subnetworks, rich-club networks and peripheral networks outside the rich club.

## 2 Methods

### 2.1 Subjects

Seven patients diagnosed with drug–resistant epilepsy of the right temporal lobe (rTLE, 4 males, mean age 31.8, range 19–50, 6 right handed, 1 left handed, for heterogeneity we selected only right lateralized patients, for detailed information see Supplementary material 1) and fourteen healthy subjects with no history of neurological disease were recruited into the study. The participants signed an informed consent form according to the rules of the local ethics committee (Comité de Protection des Personnes (CPP) Marseille 2). One control was excluded due to excessive head motion resulting in diffusion artifacts (resulting in a final control group of 13, mean age 31.8, range 20–59, 7 males, 12 right handed, 1 ambidextrous).

### 2.2 MRI acquisition

The participants were scanned on a Siemens Magnetom Verio 3 T MR-Scanner (Siemens, Erlangen, Germany). 350 functional MRI images were acquired in a BOLD-sensitized EPI T2*-weighted sequence with a TR of 3.6 s (2.0 × 2.0 × 2.5 mm, TE = 27 ms, 50 slices, FA = 90°), resulting in a total fMRI time series of 20 min. During the resting state protocol the subjects were asked to keep their eyes closed and not to fall asleep. The dMRI-sequence was acquired with the following parameters: angular gradient set of 64 directions, TR of 10.7 s (2.0 × 2.0 × 2.0 mm, TE = 95 ms, 70 slices, b weighting of 1000 s/mm^2^). T1-weighted anatomical images were acquired with a MPRAGE-sequence (TR = 1900 ms, TE = 2.19 ms, 1.0 × 1.0 × 1.0 mm, 208 slices).

### 2.3 Diffusion MRI preprocessing

To correct for head motion effects, the gradient direction matrix was rotated using a customized in-house algorithm (Leemans and Jones, 2009, Raffelt et al., 2012). Next, to reduce spatial intensity inhomogeneities, bias correction was performed on the b0 image and subsequently applied to all other diffusion volumes (Sled et al., 1998). Lastly, a Higher Order Model Outlier Rejection model (Pannek et al., 2012) identified voxels with residual outliers in the diffusion-weighted signal.

### 2.4 Fiber tracking

The fiber orientation distribution (FOD) function was estimated within MRtrix software (Tournier et al., 2012), by performing constrained spherical deconvolution (CSD, *lmax* = 8) of the diffusion signal (Tournier et al., 2008) within single-fiber (FA > 0.7) populations. The probabilistic streamline algorithm (iFOD2) (Tournier et al., 2010) was employed to reconstruct 2.5 million whole-brain fiber tracks of high-angular resolution. As streamlines were generated from random seeds throughout the brain volume, two tractography runs (i.e. 2 × 2.5 million) were performed to ensure comprehensive brain coverage. A set of plausible fiber trajectories are generated by iFOD2 by random sampling from the orientation likelihood inherent in each FOD along each candidate path. The default tracking parameters were employed for the acquisition parameters of the present study (step size = 1.25 mm, minimum length = 20 mm, max length = 200 mm, FOD termination threshold = 0.1, curvature constraint = 1 mm).

### 2.5 Structural connectome construction

Anatomical nodes of interest were generated by parcellating the standard AAL template (Tzourio-Mazoyer et al., 2002) into 512 cortical and sub-cortical regions (Supplementary material 2, see also (Perry et al., 2015)) of approximately uniform size (Zalesky et al., 2010b). In order to transform parcellations into subject-space the parcellated template was first co-registered to the MNI T1 2 mm brain template. The parcellated template (in MNI space) was subsequently transformed into subject-space by applying the transformation matrix generated from registering the MNI template to the subject's FA image. All co-registrations were affine linear registrations (to better conserve uniform regions size we did not use non-linear transformations) employed within the FSL software package (FSL 5.0 FLIRT, http://fsl.fmrib.ox.ac.uk/fsl/fslwiki/, (Smith et al., 2004)).

Within a weighted graph *G*_*w*_, a weighted connection *w*_*ij*_ represents the number of streamlines from region *i* terminating within *j*. Because we want to use Euclidian distance as a covariable in our later analysis, here we do not divide the tract count by distance (Cheng et al., 2012). For the resulting number of streamlines of the two tractography runs we excluded every connection *w*_*ij*_ with zero streamlines in at least one run or a difference of streamlines larger than three standard deviations (according to the variation across the whole individual connectome) between the two runs. This resulted in an individual structural connectome matrix with average sparsities of 0.46 (control group) and 0.44 (rTLE group, control-patient difference non-significant p > 0. 05, Wilcoxon rank-sum test). Our main interest is to keep a maximum of information for measurement of whole brain structural-functional relationship so further thresholding is only applied were absolutely needed (see below).

### 2.6 fMRI preprocessing

The T2*-weighted MRI volumes were corrected for movements and slice timing using the SPM8 toolbox (http://www.fil.ion.ucl.ac.uk/spm/software/spm8/). The subdivided AAL atlas (512 regions, without cerebellum, see above) was linearly transformed into the subjects T1-image space (FSL 5.0 FLIRT). Then the T1 volume and regional parcellation were coregistered with the T2* image (SPM8). For each time point all voxels in one region were averaged, followed by regressing out head movement parameters (six parameters, calculated during the movement correction step), cerebrospinal fluid (CSF) signals, white matter signals (both extracted from a manually selected 5 mm spherical ROI; Marsbar Toolbox 0.43, http://marsbar.sourceforge.net/) and global mean signal according to (Goñi et al., 2014, Honey et al., 2009, van den Heuvel et al., 2013). The motion parameters framewise displacement (FD) and DVARS proposed by Power et al. (2012) did not differ significantly between groups (*t*-test, p(FD) = 0.12, p(DVARS) = 0.11).

### 2.7 Functional connectome construction

We used wavelet analysis on the resulting region-averaged time series (Brainwaver toolbox, version 1.6, http://cran.r-project.org/web/packages/brainwaver/index.html) keeping only the time series of wavelet coefficients of the second wavelet scale which represent the frequency band from 0.03 Hz to 0.07 Hz (Achard et al., 2006) for a TR of 3.6 s. Finally we created a functional connectivity matrix by calculating the Pearson-correlation between each region's wavelet coefficient time series.

### 2.8 Network based statistics analysis, graph analytical measures and rich club analysis

#### 2.8.1 NBS

To compare the functional connectivity matrices of the two groups, we used network based statistics (NBS), a statistical test to correct for multiple comparisons in a network which generates a p-value by comparing network sizes of permuted random group samplings (Zalesky et al., 2010a). Correlations and anticorrelations between regional BOLD signals can both be regarded as a functional connection (Achard et al., 2006). To measure the functional connectivity strength we thus took the absolute correlation value for statistical testing. According to literature the single-connection t-value threshold was arbitrarily set sufficiently high (t ≥ 5) to avoid problems raised by low t-thresholds (as discussed for t < 4 by Smith and Nichols (2009)). The corrected p-value of NBS was calculated by finding the maximum network size of connectivity changes between 10,000 times randomized group labels (networks with p < 0.05 were considered to be significant, NBS implementation of the ConnectomeViewer toolbox (V2.0.0): http://cmtk.org/viewer/).

#### 2.8.2 Graph analytical measures

**Fig. 1:**
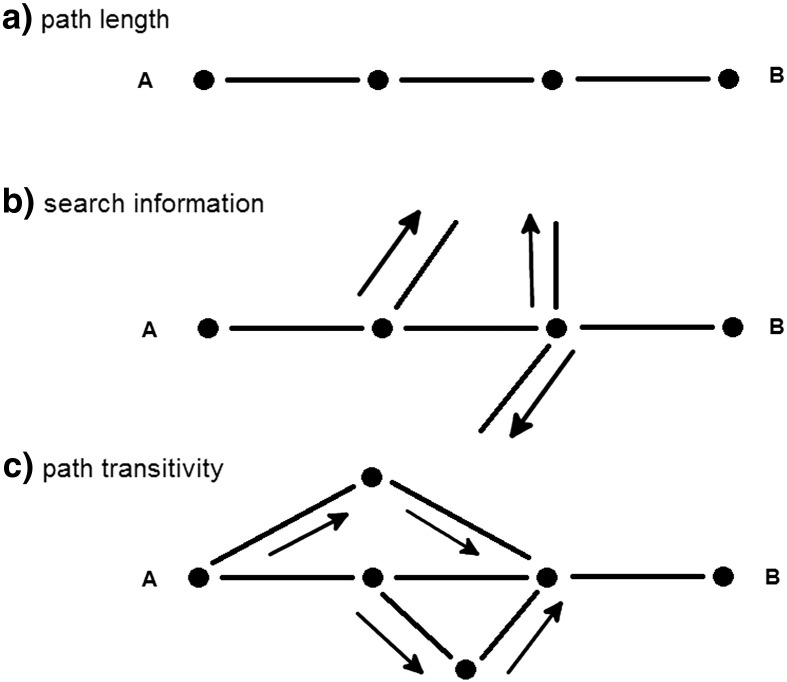
Illustration of graph metrics to characterize features of the shortest path from A to B: a) weighted path length (summed connection strengths); b) search information (weight path length by paths branching off in one of the shortest path’s nodes) and c) path transitivity (path lengths weighted by additional triangle detours which can be used to arrive at B).

To analyze the topological properties of structural connectivity we used the weighted path length (the inverse of streamline count); search information (a metric which penalizes the shortest path by the number of edges branching off in any of the nodes along the path); and path transitivity (a metric taking into account the weight of the shortest path and all paths having one supplementary step compared to the shortest path to reach the target region). Path length, search information and path transitivity metrics are illustrated in Fig. 1. Detailed formulae can be found in (Goñi et al., 2014).

In addition to graph analytical predictors of functional connectivity we defined the following metrics: Euclidian distance (distance in mm between regions centers), streamline count and an analytic prediction of functional connectivity (FCA) (Robinson et al., 2014). The FCA was defined by a linear model estimating the edgewise covariance *Cov* from the structural weight matrix *W* by

(1) $$\mathrm{Cov}={\left(-I+\mathrm{cW}\right)}^{-1};$$

with *I* being the identity matrix with the same size of the weight matrix *W* and *c* being the free coupling parameter. FCA of the covariance matrix is obtained by transforming the edgewise covariance estimates into correlation values:

(2) $$\mathrm{FC}A_{\mathrm{ij}}=\frac{\mathrm{Co}v_{\mathrm{ij}}}{\mathrm{Co}v_{\mathrm{ii}}\mathrm{Co}v_{\mathrm{jj}}};$$

The coupling parameter c was defined in respect to the maximal correlation between FCA derived from the averaged structural connectivity and averaged functional connectivity of the control group (c = 0.32). This coupling parameter was also applied to calculate the FCA of the rTLE group.

#### 2.8.3 Rich-club analysis

To compare whole brain differences the averages over the whole structural connectivity matrix of each metric were compared between the groups (Wilcoxon rank-sum test). The rich club is a high strength core of hubs which are more densely connected to each other than expected by chance. To extract the rich club of each group we used the group averaged structural connectome. To exclude effects from varying sparsity on rich club analyses (Ercsey-Ravasz et al., 2013) and to have a sparsity comparable to earlier rich club studies (van den Heuvel et al., 2013) we limited the connections of each subject to the 30% strongest connections in the structural connectome. The weighted rich club coefficient is defined as:

(3) $$\phi^{w}\left(k\right)=\frac{W_{>k}}{\sum_{l=1}^{E_{>k}}w_{l}^{{\mathrm{ranked}}^{*}}};$$

where k refers to the nodal degree; *W*_>* k*_ the summed weights of all links greater than the degree threshold k; *E*_>* k*_ is the number of links in the node subset given by threshold k, and; *w*^*ranked*^ the vector of the ranks of all weights in the observed network having a degree greater than k. Thus *ϕ*^*w*^(*k*) is the ratio between the summed weights and the sum of ranks of each link in the subset thresholded by k (see (Opsahl et al., 2008, van den Heuvel and Sporns, 2011) for details).

A rich club coefficient for each averaged connectome was calculated and normalized according to the rich club coefficient distribution of 10,000 random networks with equivalent nodal degree distributions as implemented in the brain connectivity toolbox (https://sites.google.com/site/bctnet/, version: bct-cpp r396) (Rubinov and Sporns, 2010). We characterized the profiles of normalized rich club coefficients according to a sliding degree threshold (150 < k < 350) and extracted rich club networks at a minimum degree threshold of 250, 300 and 330 connections. The lower border was selected for comparability to previous descriptions of rich club regions (van den Heuvel and Sporns, 2011). The upper limit was defined by the maximal degree of the two connectomes.

To better understand the robustness of control and patient subnetworks at high thresholds we gradually increased the threshold k and compared the size of each subnetwork in patients and controls, from which it was possible to obtain a *p* value by permuting of group labels 10,000 times.

### 2.9 Role of lesions in structural and functional connectome

Previous work has shown that network organizations in TLE patients can differ depending on the presence of brain lesions for both structural and functional connectivity (Campos et al., 2015, Coan et al., 2014). To rule out the possibility that the network reorganization observed here are caused by the underlying lesion type we show that search information is augmented even in patients with normal MR (Supplementary material 3). Additionally we validated the observed functional NBS subnetwork by showing that almost all connections in the subnetwork in every subject represented higher correlations than the control mean (except five individual connections which were found to be smaller in different subjects), with no differences for lesional or non-lesional patients (Supplementary material 3).

### 2.10 Correlation of structural and functional networks

We defined structure-function relationship as the Pearson correlation between functional connectivity and the various metrics derived from the underlying structure. To analyze the structure-function relationship we correlated these edgewise metrics with edgewise functional connectivity. This network communication approach has been shown to describe the structure-function relationship through high correlations between structural and functional connectivity (Goñi et al., 2014). Correlation of the Euclidian distance enables the analysis of gradual change in functional connectivity as a function of topological distance representing structural change accumulating along the streamlines particularly in long-range highly myelinated structures not quantified by streamline count. Accounting for distance is also necessary to control for the strong relationship between tract length and tract count (Ercsey-Ravasz et al., 2013). Correlation with analytical functional connectivity (FCA) can be used to estimate functional connectivity between regions with no direct structural connections. This enables us to identify the main factors of structure-function relationship organization in rTLE. To better predict functional connectivity we averaged all edgewise metrics over each group and correlated them with the averaged functional data (Goñi et al., 2014, Honey et al., 2009). To test for differences in correlation values and the slopes of the linear regressions we permuted group labels and recalculated mean group connectomes 10,000 times. Additionally to these metrics we tested stepwise multi-parametric linear regression models based on combined streamline count, path length, path transitivity and search information with and without accounting for the Euclidian distance.

Finally, we systematically applied streamline count, search information and Euclidian distance predictors from our previous analysis to investigate structure-function relationship in four subnetworks: an NBS-derived network of altered functional connectivity; and three structurally defined networks in the form of the rich club, feeder and peripheral/local networks. These structural networks were classified into three different classes according to its relationship to the rich club (at the selected degree threshold of k > 250, determined by best fit with previous rich club work (van den Heuvel and Sporns, 2011)). The first class consists of connections interconnecting rich club regions, the second class consists of feeder connections connecting rich club regions with non-rich club regions and the third class consists of peripheral local connections connecting non-rich club nodes with other non-rich club nodes.

## 3 Results

### 3.1 Functional connectivity in rTLE

**Fig. 2:**
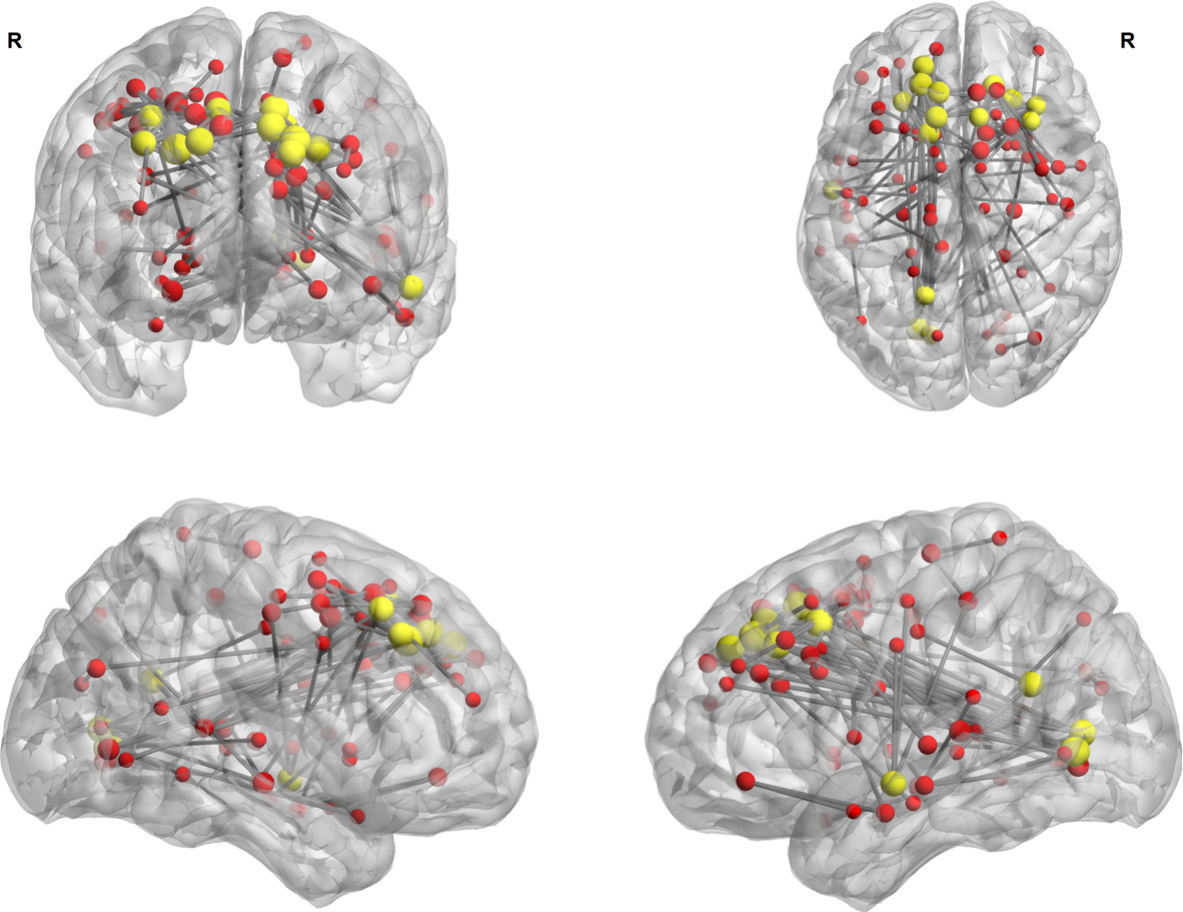
Network based statistics (NBS) on absolute functional connectivity (t-test rTLE > controls, subnetwork of modified edges threshold T > = 5, p (NBS corrected) < 0.05). Yellow nodes represent a subnetwork hub at nodal degree k > = 5 (considering only subnetwork connections), red nodes represent a degree k < 5.

NBS analysis of the functional connectome revealed a widespread hypercorrelated network defined through absolute values of correlation coefficients which were significantly higher in the rTLE group than in controls (NBS parameters: t ≥ 5, p(corrected) ≤ 0.05, Fig. 2, Supplementary material 4). Main parts of the network consist of fronto-occipital and fronto-temporal connections with a dominance of connected nodes in the left hemisphere both connected inter and intra hemispheric.

Hubs of the altered NBS subnetwork (degree ≥ 5 considering only subnetwork connections) were bilateral middle frontal gyri, superior frontal gyri and bilateral supplementary motor area, left lingual gyrus, precuneus, superior medial gyrus, middle temporal gyrus and left calcarine sulcus. Connections between those hubs can be split into a short-ranged dense locally-connected frontal network and a widespread long range connected network with the strongest connection between bilateral frontal lobes to bilateral lingual gyri but also to the right superior temporal gyrus and left middle temporal gyrus. Additionally we observed a smaller altered network of long-range connections between bilateral hippocampi, the left precuneus, thalamus and left calcarine sulcus (Fig. 2).

Non-hubs of the altered NBS subnetwork (degree < 5 considering only subnetwork connections) were located in bilateral hippocampi, fusiform gyri, putamen, rectus gyri, superior temporal gyri, precentral gyri, postcentral gyri, cuneus, occipital gyri and bilateral medial cingulum as well as in the right lingual gyrus, right calcarine sulcus, middle temporal gyrus, left thalamus, superior parietal gyrus, paracentral gyrus and left anterior cingulum (Fig. 2).

### 3.2 Structural connectivity in rTLE

The mean values of the streamline counts, path length, FCA, path transitivity and mean structural nodal degree did not differ significantly between the two groups (Supplementary material 1). Only average search information differed significantly between the controls and rTLE patients (p = 0.007 Bonferroni corrected, Wilcoxon rank sum test).

**Fig. 3:**
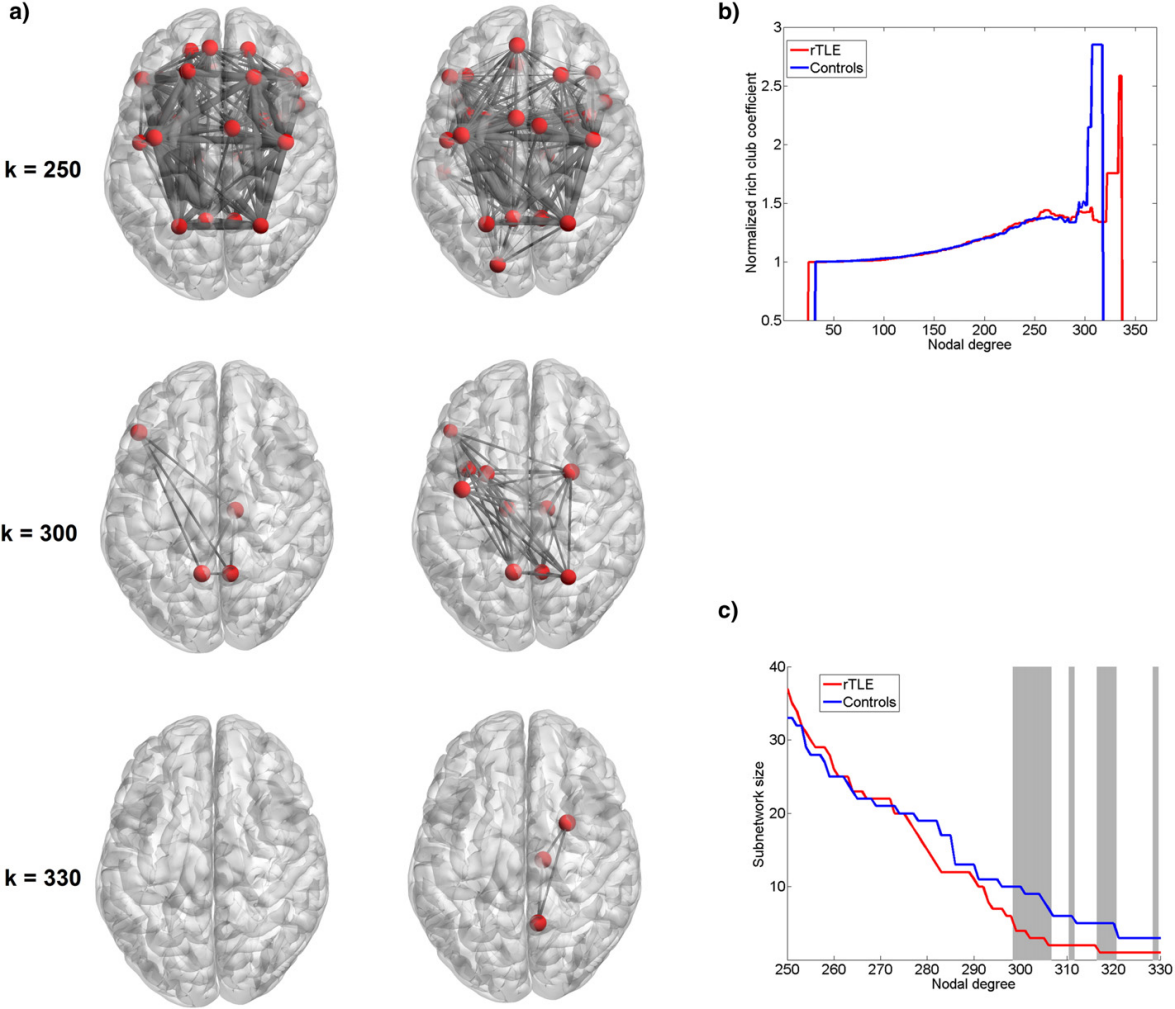
a) High degree rich club nodes and their intra-connections at a degree threshold of k = 250, 300 and 330 connections for controls and rTLE patients forming a rich club at different levels (for better visualization 512 regions are projected on the 90 regions of the AAL atlas); b) Rich club coefficient for averaged connectomes (connectome sparsity = 30%, rich club coefficient weighted by streamline counts); c) Subnetwork size (number of nodes) of the largest component as a function of the nodal degree threshold (connectome sparsity = 30%), grey background marks significant difference between the two groups (p < 0.05, two-sided t-test, 10000 permutations of group labels to build the averaged connectome).

At a degree threshold of 150–250 we observed mostly equivalent regions in patients and controls and a similar normalized rich club coefficients profile (see Fig. 3a, b. Fig. 3c shows the rich club subnetwork size as a function of the nodal degree threshold to define this subnetwork for both controls and rTLE patients. From a degree threshold of 300 connections rTLE patients showed a significantly larger rich club subnetwork than observed in controls. The rich club started to disintegrate at a degree threshold of 300 for the control group and at a threshold of 330 for rTLE patients. Fig. 3a shows rich club regions at different nodal degree thresholds of 250, 300 and 330.

At a degree threshold of 250, rich club regions for controls included bilateral precentral gyri, superior frontal gyri, superior orbital frontal gyri, insulas, superior parietal gyri, precuneus and bilateral putamen, right superior temporal pole and right support motor area, left inferior triangularis and left inferior orbital frontal gyrus. At the same degree threshold of 250 for rTLE patients, the rich club did not include the left superior frontal gyrus and the bilateral orbital frontal gyri, but rather the left hippocampus, left superior temporal pole, left rolandic operculum, left supplementary motor area and right calcarine were part of the rich club.

At a degree threshold of 300, the rich club of controls included bilateral precuneus, right thalamus and left inferior frontal triangularis. For rTLE patients, the rich club was composed by bilateral precuneus, putamen and bilateral thalami, left precentral gyrus, inferior frontal triangularis and insula and right superior parietal gyrus.

At a degree threshold of 330 no regions could be considered to belong to the rich club in controls while in rTLE patients, rich club properties were found for the right precuneus, putamen and thalamus. Thus, at this highest degree threshold, key regions form a rich club in only patients.

### 3.3 Structure-function relationship in rTLE

Regression of using each structural connectivity metric as a single predictor (streamline count, FCA, path length, path transitivity, search information, Euclidian distance) showed significant correlations with functional connectivity (P < 10^− 6^, Table 1). Euclidian distance had the highest correlation values both in control and in rTLE patients. Multiparametric stepwise regression including all graph analytical parameters (streamline count, path length, path transitivity, search information and Euclidian distance) showed that each of the parameters contributed significantly to the model (P < 10^− 6^).

**Fig. 4:**
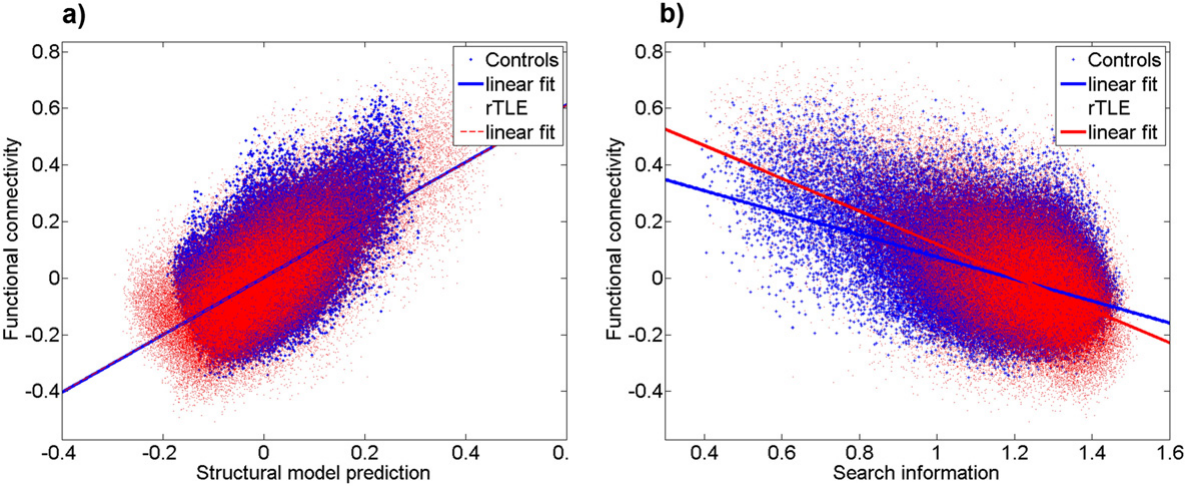
Prediction of functional connectivity a) using a combined model of euclidian distance, path length, path transitivity and search information (contols R = 0.575, rTLE patients R = 0.656, rTLE > controls: p < 0.05, 10000 permutations ) and b) using group averaged search information (controls R = −0.46, rTLE patients R = −0.535, rTLE > controls: p < 0.05, 10000 permutations).

Comparing the relative strength of the structure-function relationship between controls and rTLE patients using permutation testing showed significant differences (10,000 permutations, p < 0.05) in correlation for all metrics and significant differences for slope values of simple regression models for FCA, path transitivity, search information, Euclidian distance (Table 2, Fig. 4).

**Fig. 5:**
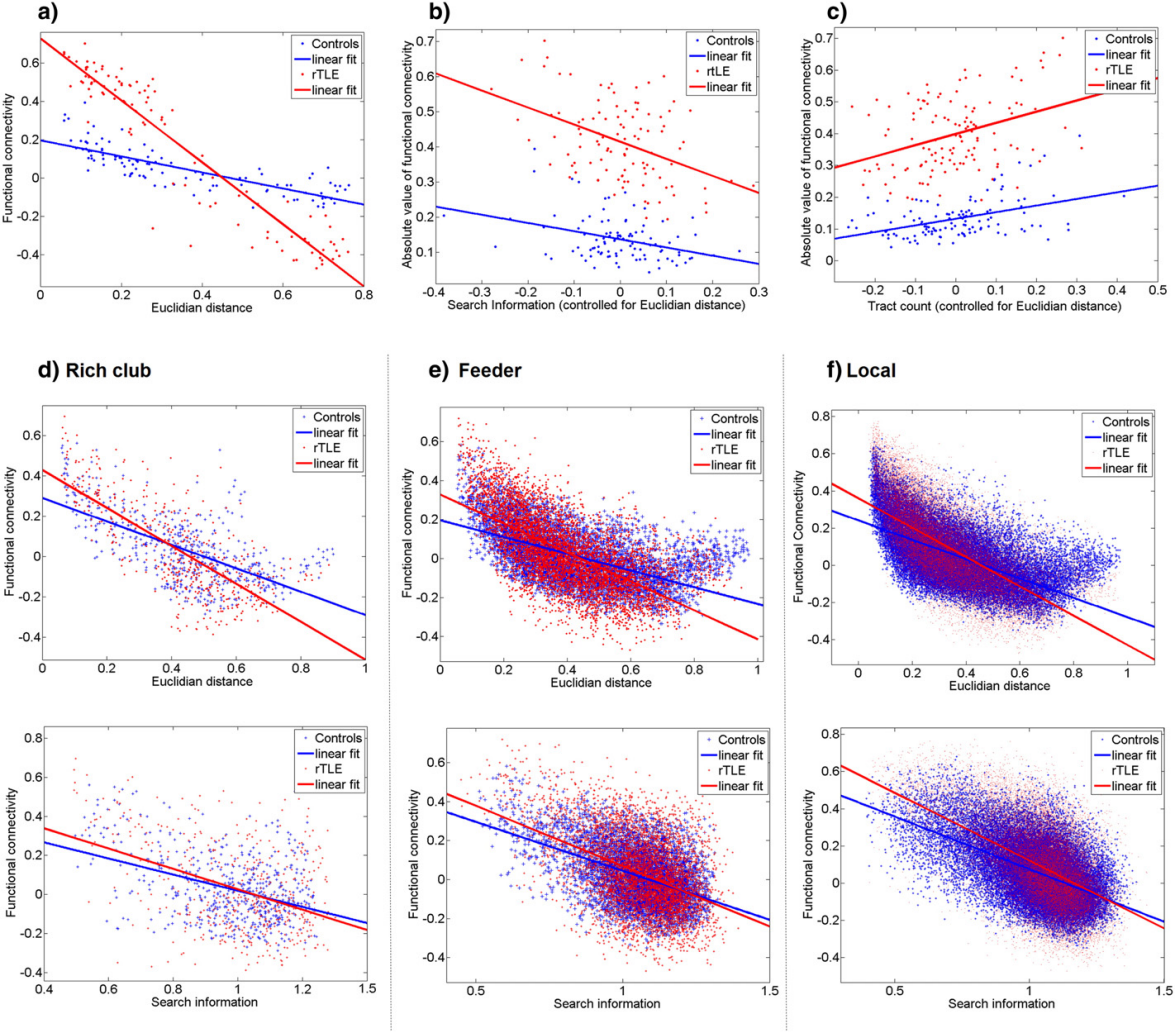
Prediction of functional connectivity for controls and rTLE patients using a) Euclidian distance, b) search information and c) streamline count based on group averaged connectomes for connections in the altered NBS subnetwork of functional connectivity; Euclidian distance (middle row) and search information (lower row) vs. functional connectivity in three subnetworks at a degree threshold k > 250 splitting the brain in d) Rich club connections, e) Feeder connections and f) Local connections. For detailed correlation values and slope differences see Table 1, Table 2.

The altered functional NBS subnetwork comparing rTLE and controls showed higher structure-function correlation for all metrics (Table 1) compared to the whole brain analysis. The Euclidian distance was also the best predictor of structure-function relationship but contributions of search information and streamline count remained significant when controlling for Euclidian distance (Fig. 5). The rich club edges had showed an increased correlation for Euclidian distance and path length, while the other metrics decreased in comparison to the whole brain analysis. The streamline count was more highly correlated in the peripheral network may be because of the use of a fixed sparsity of 30% which excludes connections with streamline counts of zero, in contrast to the more inclusive whole brain analysis. Correlation of the other metrics was lower than in whole brain analysis.

Slopes of the linear fit differed between the two groups for the altered NBS subnetwork and the peripheral network. Neither the rich club nor feeder connections showed a different slope of structure-function correlation between controls and rTLE patients (Table 2). We did not estimate multiparametric models because both the altered NBS subnetwork and the rich club subnetwork lacked sufficient data to obtain significance of all regression coefficients in the model (P < 0.001).

Looking at structure-function correlation differences between rTLE patients and controls within the rich club, feeder and peripheral networks respectively the only significant difference was observed for peripheral connections (Fig. 5).

## 4 Discussion

In this study we systematically investigated the descriptive and explanatory power of graph theory and network communication measures to characterize the structure-function relationship in the normal and epileptic brain. By focusing on right lateralized TLE we emphasized homogeneity across patients to allow for a maximum of comparability of our findings. In rTLE patients, on a whole brain level we observed a general pattern of increased functional connectivity, increased global search information, a preserved rich club, as well as an increased structure-function correlation for all metrics compared to healthy controls. Notably the prediction of functional connectivity by structural parameters such as Euclidian distance and search information capture structure-function reorganization in rTLE. Compared to peripheral connections the structurally preserved rich club shows no differences in structure-function relationship.

### 4.1 Reorganization of the functional connectome in the rTLE group

NBS-analysis revealed a widespread hypercorrelated network for rTLE patients in comparison to controls mainly consisting of fronto-occipital and fronto-temporal connections. This altered NBS subnetwork overlapped with default mode network regions such as the precuneus and precentral gyrus, middle temporal gyrus and the temporal pole with more connections in the left hemisphere. This is in line with previous whole brain pairwise analysis of rTLE which also found connectivity increases in the left hemisphere (Su et al., 2015). Though concerned only with pairwise connectivity data the current work is in line with previous graph theoretical demonstrations of topological rewiring in temporal lobe epilepsy and widespread functional connectivity changes for ipsi- and contralateral temporal lobe and default mode network regions for rTLE patients (Liao et al., 2010, Voets et al., 2012). Our results differ regarding the previously found decreases of connectivity (Liao et al., 2010, Su et al., 2015). A possible explanation is that our results are not directly comparable to previous work because we took absolute connectivity values to treat account for positive and negative correlations (as these both represent an inter-regional coupling) in a homogeneous manner.

Previously we found increased functional connectivity outside the epileptogenic zone correlated to altered memory performance (Bettus et al., 2009) suggesting a global reorganization of functional connectivity in TLE. This increased synchronization of BOLD signals could be an expression of more homogenous brain functioning with a decrease in functional repertoire (Maccotta et al., 2013) caused by strong self-sustaining oscillatory states induced by an increased noise level (Deco et al., 2009) due to epileptic activity. In line with this, we previously found a homogenization of hubs by flattening the differences between hubs and non-hubs (Ridley et al., 2015). This might be another consequence of the here observed globally decreased functional repertoire. Of note, these increased correlations did not involve functional connectivity *between* regions commonly implicated in the individual epileptogenic network in rTLE patients (Bettus et al., 2009) but relates to edges *terminating* in core epileptic nodes such as the right hippocampus (See right temporal lobe, Fig. 2).

### 4.2 Alteration of the structural connectome in the rTLE group

Among the different parameters we used to characterize structural connectivity (streamline count, FCA, path length, search information, path transitivity), only the search information metric (averaged over the whole brain) was shown to be different between rTLE patients and controls. Previous whole brain structural studies found decreased path length in TLE patients (see (Haneef and Chiang, 2014) for review), by either exploring a range of arbitrary sparsity thresholds of the connectivity matrix (Bernhardt et al., 2011) or by restricting the sparsity artificially through building consensus networks of largely heterogeneous groups of temporal and frontal lobe epilepsy (Vaessen et al., 2012). Our approach was to retain only connections varying less than three standard deviations in the two tractography runs. This resulted in a sparsity of 0.45. In line with (Bernhardt et al., 2011) path length does not differ at this sparsity. Nevertheless our search information parameter was shown to be sensitive to rTLE with no need to arbitrarily adjust the matrix sparsities.

Conversely NBS-corrected pairwise connections on a global level using streamline count were not different, concordant with findings at the whole brain scale in other studies in rTLE (Bonilha et al., 2012). This suggests search information as a plausible new parameter to measure global scale reorganization of the structural network in rTLE. These results are in line with recent findings stressing a global network involvement with loss in white matter integrity and rise in connections at the local scale (Bonilha et al., 2012) as well as at the whole brain scale (DeSalvo et al., 2014). Investigations of histology have provided evidence of abnormal myelinated cortical fiber tracts in the temporal lobe (Thom et al., 2000), altered white matter neuronal density (Thom et al., 2001) and widespread white matter alterations (Blanc et al., 2011) that might trigger the observed modified structural and functional connectivity in TLE.

Structural brain lesions in many brain disorders such as TLE are more likely to occur in hub regions of the human brain (Crossley et al., 2014). By extracting the rich club coefficient to examine nodal degree changes in central interconnected hubs, we observed a rich club organization in controls and rTLE patients consistent with the high resolution rich club described in van den Heuvel and Sporns (2011). Comparison of rich club coefficients (van den Heuvel et al., 2013) between rTLE patients relative to controls did not show any differences.

Despite this strong overlap an increased nodal degree threshold reveals a differing disintegration of the rich club network showing different core components between rTLE patients and controls. When increasing the nodal degree thresholds, resilience of the rich club network was higher for rTLE patients relative to controls. Interestingly, among nodes of networks that are divergent between rTLE and controls at higher degree thresholds, the hubs constituting the rich club included bilateral thalami, which have been shown to be i) highly involved during seizures in TLE (Guye et al., 2006), ii) prone to atrophy (Bernhardt et al., 2012, Keller et al., 2014) and iii) prone to topological reorganization (Bonilha et al., 2012). In addition, thalamo-parietal connectivity has previously been found to play a central role in seizure propagation in TLE (Arthuis et al., 2009). Bonilha et al. (2012) previously reported increased nodal degree of insula and bilateral thalami. Structural changes in the putamen have been previously found in DTI studies (Keller et al., 2013) and furthermore the parietal lobe, thalamus, posterior cingulate gyrus/precuneus and insula were shown to be implicated during TLE seizures (Bartolomei et al., 2001, Gotman et al., 2005, Kahane and Bartolomei, 2010).

It is important to note that the observed increased streamline count does not automatically imply an increase in anatomical fibers when comparing patients to controls. The tractography algorithm employed here which uses a fixed number of streamlines cannot discern whether the more highly connected regions of the rich club in patients reflect genuinely augmented degree in those specific regions in comparison to controls, or are merely the regions with preserved degree despite a global fiber loss (Calamante et al., 2015). This point can be resolved by taking into account structure function alterations of these rich club regions which are not influenced by the limitation of constant streamlines (see Section 4.5). Given the role these regions are known to play in epilepsy, the notion of a preserved rich club despite widespread fiber loss could explain disease symptoms by providing a propagation channel of epileptic activity through this preserved network.

### 4.3 Structure-function relationship in the whole brain

In line with previous work in healthy controls, we observed significant correlation between functional connectivity and structural parameters such as Euclidian distance and streamline count (Honey et al., 2009). Furthermore, we demonstrated the high predictive value of the search information metric in relation to functional connectivity similar to previous reports in healthy brains (Goñi et al., 2014). The multivariate analysis showed the significant added value of Euclidian distance in the prediction of functional connectivity when added to the regression model of search information, streamline count, path length and path transitivity.

By relating structural to functional alterations, this graph analytical approach could help deciphering discrepancies in results between structural and functional low-level connectional disruptions in TLE (Bernhardt et al., 2013). In contrast to results obtained in generalized epilepsy showing a structure-function decorrelation (Zhang et al., 2011), we observed in this group of rTLE patients an increased structure-function correlation. This might be due to the different phenotypes of disease. Interestingly, idiopathic generalized epilepsy is less characterized by cognitive dysfunction and particularly social cognitive dysfunction than TLE Epilepsy (Realmuto et al., 2015), which suggests different underlying reorganizations of structure and function between the two phenotypes.

From a pathophysiological point of view, differences between rTLE patients and controls in the prediction strength of the Euclidian distance metric with regards to functional connectivity might be related to altered cumulative structural damage along the streamline (e.g. gliosis or demyelination (Thom et al., 2000)) that did not change the fiber orientation. This observed altered distance-function correlation might reflect changed time-lag structure in functional resting-state connectivity in rTLE (Mitra et al., 2015). In addition, while we observed increased search information in patients and its ability to better predict functional connectivity, the mean path transitivity does not differ between the two groups. This suggests a reorganization of networks in rTLE patients via additional, spurious connections acting as branches off the shortest path. Increased search information might be also linked to previously found decreased global fractional anisotropy (Bernhardt et al., 2013) which would cause the tractography algorithm to find more secondary streamlines branching off the main tract. Especially for directly connected nodes this can add useful information for prediction of functional connectivity in opposition to weighted path length which reflects only the inverse streamline count for directly connected nodes. Like the earlier discussed increased functional connectivity, augmented structure-function correlation suggests more homogeneous functional connectivity in rTLE approaching the underlying structural connectivity, which might represent a loss in the functional repertoire of the rTLE brain.

### 4.4 Structure-function relationship in the hypercorrelated functional network identified by NBS

Connections of the altered functional NBS subnetwork between controls and rTLE patients had higher structure-function correlations than observed for whole brain analysis (both controls and patients). This suggests that the functional changes of epilepsy predominantly manifest in brain subnetworks with pre-existing strong structure-function coupling in controls. This relationship was observed for both Euclidian distance and tractography-(derived) measures like streamline count and search information (Fig. 5). Notably the effect for streamline count and search information persists even when we control for Euclidian distance. Interestingly in contrast to whole brain analysis we found that after regressing out the distance, streamline count predicts functional connectivity better than search information (while both metrics remain significantly correlated to functional connectivity).

### 4.5 Structure function relationship in rich club and peripheral regions

Function-structure correlations observed in the rich club, feeder and peripheral connections (Fig. 5d–f) revealed correlation changes in peripheral connections only. This might be in relation to observations in macaque data attributing slower dynamics to the rich club compared to the decentralized periphery (Gollo et al., 2015), suggesting that variation due to dynamic pathological epileptic activity will also be more likely to be observed in the peripheral network given the relative scarcity of additional stabilizing connections as found in the rich club. Taking into account the rich club alterations (see Section 4.2) - which could be linked either to a preserved core in the presence of global fiber loss or a hyperconnected core in an unchanged connectome, this result is more in favor of the latter alternative. This is supported by previous studies which found localized but widespread structural degeneration in rTLE (Bernhardt et al., 2013, Besson et al., 2014). This is also in line with previous findings of rich club preservation in the elderly brain (Perry et al., 2015) and in Alzheimers disease (Daianu et al., 2015). Taking into account the general degenerative progression of epilepsy (Avanzini et al., 2013, Goddard et al., 1969) this preservation of the central core could possibly be a necessary mechanism to prevent a collapse of global brain functioning at the price of constant added noise by the epileptogenic zone. Future work should validate if this preserved structural functional network indeed eases spreading of seizures, for example through hub nodes such as thalamus, insula or precuneus. This might provide new insight on the characterization of epilepsy as a functional disease of uncontrolled information spreading in a closed network with secondary degenerative effect on the structural connectome. In the light of these results it would be especially interesting to better understand the possibility of brain adaptation to decrease potential seizure propagation through this central core limited by preserving a general functioning of the brain.

### 4.6 Structure-function relationship and non-stationary brain dynamics

It should be noted that in the current paper we have considered structure function correlation as averaged over a relatively large acquisition period (20 min). Previous literature (Honey et al., 2007) would lead us to expect that under this paradigm, variation through time as the brain oscillates through various states will be averaged out and thus the functional connectome approaches the underlying structural connectome. The importance of the current work is that structure-function correlation is augmented in epileptic patients. However, recent studies pointed out that the time averaged correlation of the fMRI is an oversimplification of resting state fMRI data (Allen et al., 2014, Hansen et al., 2015, Tagliazucchi and Laufs, 2015, Zalesky et al., 2014). In particular, time averaged data may suppress non-stationarities in the dynamic behavior of networks. In this context, the approach taken by Deco et al. (2013) and incorporated into the FCA methodology used here is insufficient to capture the vagaries of structure-function correlation as the brain transitions between states over time. A range of possible brain organization states has been identified with the brain's dynamic repertoire (Deco et al., 2011). The tighter coupling observed here in rTLE patients between analytical structural models and functional connectivity may reflect a reduced dynamic repertoire of possible brain states which could be observed via Functional Connectivity Dynamics (FCD) (Hansen et al., 2015). Future time-resolved dynamic analyses are needed to examine the extent of structure-function augmentation in brain states resolved over smaller analysis windows.

### 4.7 Limitations

We acknowledge the limits of the sample size, chosen in order to have a relatively homogeneous group in terms of epileptogenic network location, including only right lateralized epileptic patients. Nevertheless we have shown that our data is in line with both controls (Goñi et al., 2014) and rTLE findings (Bernhardt et al., 2013). Furthermore, the use of connectomes averaged over the whole group – as per the approach of Goñi et al. (2014) – is not optimal especially in terms of the potential clinical applicability of the results. Future work should focus on deriving reliable brain network descriptors for single subject analysis of structure-function correlations. Lateralization of TLE is an important factor in both structural (Besson et al., 2014) and functional connectivity (Ridley et al., 2015) so our findings might not be generalizable to TLE.

This study cannot answer the question whether search information is a good predictor to classify rTLE structure-function relationship in general or if it is related to hub alterations generally observed in pathologic brain states (Crossley et al., 2014) and it might be interesting to apply this approach to different pathologies.

## 5 Conclusion

In this study we have demonstrated that an edge-based graph analytical approach can elucidate the impact of pathological alterations of structural connectivity on function by linking widespread hypercorrelation of networks in rTLE to structural changes. We maximized the structure-function correlation by using search information, a metric which represents the random reorganization of structural connections, revealing a significantly higher correlation in rTLE patients. Additionally we highlighted the importance of considering Euclidian distance – which is both highly correlated with structural and functional connectivity data - as a major confounding variable of functional-structural correlations in rTLE, possibly explaining structural alterations in TLE which cannot be captured by dMRI based tractography. Alterations seem to be more likely to appear in the local periphery of the brain while core connections show preserved structural and functional stability. Increased functional connectivity and increased structure-function correlation suggest a fallback to structure and loss of functional variability in rTLE patients.

The following are the supplementary data related to this article.

Supplementary material 1 Right temporal lobe patients characteristics and average connectivity metric statistics. Supplementary information 1

Supplementary material 2 Parcellation Information. Supplementary information 2

Supplementary material 3 Patient data evaluation. Supplementary information 3

Supplementary material 4 Network based statistics of functional connectivity. Supplementary information 4

## Figures and Tables

**Table 1:** Pearson correlation between functional connectivity and structural derived metrics in the whole brain and in subnetworks (all predictors were significant with P < 10^− 6^, group averaged connectivity matrices, NBS: altered NBS subnetwork of functional connectivity rTLE > controls, Rich Club, Feeder and Local: rich club, feeder and local connections for structural connectome sparsitiy 30%, and nodal degree > 250); FCA: analytic functional connectivity, ED: Euclidian distance, PL: Path length, PT: Path transitivity, SI: search information, PP path predictors (path length, path transitivity, search information).

	Whole-brain		NBS		Rich Club		Feeder		Local	
Controls	rTLE	Controls	rTLE	Controls	rTLE	Controls	rTLE	Controls	rTLE
Streamline count (log)	0.375	0.433	0.714	0.726	0.331	0.332	0.356	0.352	0.413	0.451
FCA	0.349	0.413	0.705	0.717	0.315	0.321	0.315	0.317	0.354	0.404
ED	− 0.531	− 0.619	− 0.790	− 0.912	− 0.635	− 0.748	− 0.601	− 0.676	− 0.619	− 0.676
PL	− 0.227	− 0.289	− 0.594	− 0.615	− 0.437	− 0.368	− 0.274	− 0.252	− 0.177	− 0.208
PT	0.254	0.337	0.629	0.719	0.148	0.162	0.120	0.131	0.026	0.134
SI (log)	− 0.460	− 0.535	− 0.773	− 0.833	− 0.413	− 0.425	− 0.496	− 0.482	− 0.545	− 0.559
Combined PP with ED	0.575	0.656	–	–	–	–	–	–	–	–

**Table 2:** P-values for permutation of group labels of the averaged connectome on the regression slopes of a linear fit (Controls vs. rTLE, n = 10.000, *p < 0.05); NBS: altered NBS subnetwork of functional connectivity rTLE > controls, Rich Club, Feeder and Local: rich club, feeder and local connections for structural connectome sparsitiy 30%, and nodal degree > 250), FCA: analytic functional connectivity, ED: Euclidian distance, PL: Path length, PT: Path transitivity, SI: search information, PP combined path predictors: path length, path transitivity, search information.

	Whole-brain	NBS	Rich Club	Feeder	Local
Streamline count (log)	0.0020^⁎^	0.0013^⁎^	0.3149	0.4628	0.0028^⁎^
FCA	0.0055^⁎^	0.0037^⁎^	0.3673	0.6393	0.0041^⁎^
ED	0.0111^⁎^	0.0074^⁎^	0.0935	0.0496^⁎^	0.0316^⁎^
PL	0.1942	0.0278^⁎^	0.4623	0.6588	0.2612
PT	0.0043^⁎^	0.0017^⁎^	0.4148	0.6738	0.0009*
SI (log)	0.0188^⁎^	0.0025^⁎^	0.4656	0.3579	0.0236*
Combined PP with ED	0.0019^⁎^	–	–	–	–

## References

[bb0005] Achard S., Salvador R., Whitcher B., Suckling J., Bullmore E. (2006). A resilient, low-frequency, small-world human brain functional network with highly connected association cortical hubs. J. Neurosci..

[bb0010] Allen E.A., Damaraju E., Plis S.M., Erhardt E.B., Eichele T., Calhoun V.D. (2014). Tracking whole-brain connectivity dynamics in the resting state. Cereb. Cortex.

[bb0015] Arthuis M., Valton L., Regis J., Chauvel P., Wendling F., Naccache L., Bernard C., Bartolomei F. (2009). Impaired consciousness during temporal lobe seizures is related to increased long-distance cortical-subcortical synchronization. Brain.

[bb0020] Avanzini G., Depaulis A., Tassinari A., de Curtis M. (2013). Do seizures and epileptic activity worsen epilepsy and deteriorate cognitive function?. Epilepsia.

[bb0025] Bartolomei F., Wendling F., Bellanger J.J., Regis J., Chauvel P. (2001). Neural networks involving the medial temporal structures in temporal lobe epilepsy. Clin. Neurophysiol..

[bb0030] Bernhardt B.C., Chen Z., He Y., Evans A.C., Bernasconi N. (2011). Graph-theoretical analysis reveals disrupted small-world organization of cortical thickness correlation networks in temporal lobe epilepsy. Cereb. Cortex.

[bb0035] Bernhardt B.C., Bernasconi N., Kim H., Bernasconi A. (2012). Mapping thalamocortical network pathology in temporal lobe epilepsy. Neurology.

[bb0040] Bernhardt B.C., Hong S., Bernasconi A., Bernasconi N. (2013). Imaging structural and functional brain networks in temporal lobe epilepsy. Front. Hum. Neurosci..

[bb0045] Besson P., Dinkelacker V., Valabregue R., Thivard L., Leclerc X., Baulac M., Sammler D., Colliot O., Lehericy S., Samson S., Dupont S. (2014). Structural connectivity differences in left and right temporal lobe epilepsy. NeuroImage.

[bb0050] Bettus G., Guedj E., Joyeux F., Confort-Gouny S., Soulier E., Laguitton V., Cozzone P.J., Chauvel P., Ranjeva J.P., Bartolomei F., Guye M. (2009). Decreased basal fMRI functional connectivity in epileptogenic networks and contralateral compensatory mechanisms. Hum. Brain Mapp..

[bb0055] Blanc F., Martinian L., Liagkouras I., Catarino C., Sisodiya S.M., Thom M. (2011). Investigation of widespread neocortical pathology associated with hippocampal sclerosis in epilepsy: a postmortem study. Epilepsia.

[bb0060] Bonilha L., Nesland T., Martz G.U., Joseph J.E., Spampinato M.V., Edwards J.C., Tabesh A. (2012). Medial temporal lobe epilepsy is associated with neuronal fibre loss and paradoxical increase in structural connectivity of limbic structures. J. Neurol. Neurosurg. Psychiatry.

[bb0065] Calamante F., Smith R.E., Tournier J.D., Raffelt D., Connelly A. (2015). Quantification of voxel-wise total fibre density: investigating the problems associated with track-count mapping. NeuroImage.

[bb0070] Campos B.M., Coan A.C., Beltramini G.C., Liu M., Yassuda C.L., Ghizoni E., Beaulieu C., Gross D.W., Cendes F. (2015). White matter abnormalities associate with type and localization of focal epileptogenic lesions. Epilepsia.

[bb0075] Centeno M., Vollmar C., Stretton J., Symms M.R., Thompson P.J., Richardson M.P., O'Muircheartaigh J., Duncan J.S., Koepp M.J. (2014). Structural changes in the temporal lobe and piriform cortex in frontal lobe epilepsy. Epilepsy Res..

[bb0080] Cheng H., Wang Y., Sheng J., Kronenberger W.G., Mathews V.P., Hummer T.A., Saykin A.J. (2012). Characteristics and variability of structural networks derived from diffusion tensor imaging. NeuroImage.

[bb0085] Chiang S., Stern J.M., Engel J., Haneef Z. (2015). Structural-functional coupling changes in temporal lobe epilepsy. Brain Res..

[bb0090] Coan A.C., Campos B.M., Beltramini G.C., Yasuda C.L., Covolan R.J., Cendes F. (2014). Distinct functional and structural MRI abnormalities in mesial temporal lobe epilepsy with and without hippocampal sclerosis. Epilepsia.

[bb0095] Constable R.T., Scheinost D., Finn E.S., Shen X., Hampson M., Winstanley F.S., Spencer D.D., Papademetris X. (2013). Potential use and challenges of functional connectivity mapping in intractable epilepsy. Front. Neurol..

[bb0100] Crossley N.A., Mechelli A., Scott J., Carletti F., Fox P.T., McGuire P., Bullmore E.T. (2014). The hubs of the human connectome are generally implicated in the anatomy of brain disorders. Brain.

[bb0105] Daianu M., Jahanshad N., Nir T.M., Jack C.R., Weiner M.W., Bernstein M.A., Thompson P.M. (2015). Rich club analysis in the Alzheimer's disease connectome reveals a relatively undisturbed structural core network. Hum. Brain Mapp..

[bb0110] Damoiseaux J.S., Greicius M.D. (2009). Greater than the sum of its parts: a review of studies combining structural connectivity and resting-state functional connectivity. Brain Struct. Funct..

[bb0115] Deco G., Jirsa V., McIntosh A.R., Sporns O., Kotter R. (2009). Key role of coupling, delay, and noise in resting brain fluctuations. Proc. Natl. Acad. Sci. U. S. A..

[bb0120] Deco G., Jirsa V.K., McIntosh A.R. (2011). Emerging concepts for the dynamical organization of resting-state activity in the brain. Nat. Rev. Neurosci..

[bb0125] Deco G., Jirsa V.K., McIntosh A.R. (2013). Resting brains never rest: computational insights into potential cognitive architectures. Trends Neurosci..

[bb0130] DeSalvo M.N., Douw L., Tanaka N., Reinsberger C., Stufflebeam S.M. (2014). Altered structural connectome in temporal lobe epilepsy. Radiology.

[bb0135] Douw L., DeSalvo M.N., Tanaka N., Cole A.J., Liu H., Reinsberger C., Stufflebeam S.M. (2015). Dissociated multimodal hubs and seizures in temporal lobe epilepsy. Ann. Clin. Transl. Neurol..

[bb0140] Ercsey-Ravasz M., Markov N.T., Lamy C., Van Essen D.C., Knoblauch K., Toroczkai Z., Kennedy H. (2013). A predictive network model of cerebral cortical connectivity based on a distance rule. Neuron.

[bb0145] Fornito A., Zalesky A., Breakspear M. (2015). The connectomics of brain disorders. Nat. Rev. Neurosci..

[bb0150] Goddard G.V., McIntyre D.C., Leech C.K. (1969). A permanent change in brain function resulting from daily electrical stimulation. Exp. Neurol..

[bb0155] Gollo L.L., Zalesky A., Hutchison R.M., van den Heuvel M., Breakspear M. (2015). Dwelling quietly in the rich club: brain network determinants of slow cortical fluctuations. Philos. Trans. R. Soc. Lond. Ser. B Biol. Sci..

[bb0160] Goñi J., van den Heuvel M.P., Avena-Koenigsberger A., Velez de Mendizabal N., Betzel R.F., Griffa A., Hagmann P., Corominas-Murtra B., Thiran J.P., Sporns O. (2014). Resting-brain functional connectivity predicted by analytic measures of network communication. Proc. Natl. Acad. Sci. U. S. A..

[bb0165] Gotman J., Grova C., Bagshaw A., Kobayashi E., Aghakhani Y., Dubeau F. (2005). Generalized epileptic discharges show thalamocortical activation and suspension of the default state of the brain. Proc. Natl. Acad. Sci. U. S. A..

[bb0170] Guye M., Regis J., Tamura M., Wendling F., McGonigal A., Chauvel P., Bartolomei F. (2006). The role of corticothalamic coupling in human temporal lobe epilepsy. Brain.

[bb0175] Guye M., Bartolomei F., Ranjeva J.P. (2008). Imaging structural and functional connectivity: towards a unified definition of human brain organization?. Curr. Opin. Neurol..

[bb0180] Guye M., Bettus G., Bartolomei F., Cozzone P.J. (2010). Graph theoretical analysis of structural and functional connectivity MRI in normal and pathological brain networks. MAGMA.

[bb0185] Haneef Z., Chiang S. (2014). Clinical correlates of graph theory findings in temporal lobe epilepsy. Seizure.

[bb0190] Haneef Z., Lenartowicz A., Yeh H.J., Engel J., Stern J.M. (2012). Effect of lateralized temporal lobe epilepsy on the default mode network. Epilepsy Behav..

[bb0195] Hansen E.C., Battaglia D., Spiegler A., Deco G., Jirsa V.K. (2015). Functional connectivity dynamics: modeling the switching behavior of the resting state. NeuroImage.

[bb0200] Honey C.J., Kotter R., Breakspear M., Sporns O. (2007). Network structure of cerebral cortex shapes functional connectivity on multiple time scales. Proc. Natl. Acad. Sci. U. S. A..

[bb0205] Honey C.J., Sporns O., Cammoun L., Gigandet X., Thiran J.P., Meuli R., Hagmann P. (2009). Predicting human resting-state functional connectivity from structural connectivity. Proc. Natl. Acad. Sci. U. S. A..

[bb0210] Kahane P., Bartolomei F. (2010). Temporal lobe epilepsy and hippocampal sclerosis: lessons from depth EEG recordings. Epilepsia.

[bb0215] Keller S.S., Ahrens T., Mohammadi S., Gerdes J.S., Moddel G., Kellinghaus C., Kugel H., Weber B., Ringelstein E.B., Deppe M. (2013). Voxel-based statistical analysis of fractional anisotropy and mean diffusivity in patients with unilateral temporal lobe epilepsy of unknown cause. J. Neuroimaging.

[bb0220] Keller S.S., O'Muircheartaigh J., Traynor C., Towgood K., Barker G.J., Richardson M.P. (2014). Thalamotemporal impairment in temporal lobe epilepsy: a combined MRI analysis of structure, integrity, and connectivity. Epilepsia.

[bb0225] Laufs H. (2012). Functional imaging of seizures and epilepsy: evolution from zones to networks. Curr. Opin. Neurol..

[bb0230] Leemans A., Jones D.K. (2009). The B-matrix must be rotated when correcting for subject motion in DTI data. Magn. Reson. Med..

[bb0235] Liao W., Zhang Z., Pan Z., Mantini D., Ding J., Duan X., Luo C., Lu G., Chen H. (2010). Altered functional connectivity and small-world in mesial temporal lobe epilepsy. PLoS One.

[bb0240] Maccotta L., He B.J., Snyder A.Z., Eisenman L.N., Benzinger T.L., Ances B.M., Corbetta M., Hogan R.E. (2013). Impaired and facilitated functional networks in temporal lobe epilepsy. Neuroimage Clin..

[bb0245] Mitra A., Snyder A.Z., Blazey T., Raichle M.E. (2015). Lag threads organize the brain's intrinsic activity. Proc. Natl. Acad. Sci. U. S. A..

[bb0250] Opsahl T., Colizza V., Panzarasa P., Ramasco J.J. (2008). Prominence and control: the weighted rich-club effect. Phys. Rev. Lett..

[bb0255] Pannek K., Raffelt D., Bell C., Mathias J.L., Rose S.E. (2012). HOMOR: higher order model outlier rejection for high b-value MR diffusion data. NeuroImage.

[bb0260] Perry A., Wen W., Lord A., Thalamuthu A., Roberts G., Mitchell P.B., Sachdev P.S., Breakspear M. (2015). The organisation of the elderly connectome. NeuroImage.

[bb0265] Pittau F., Grova C., Moeller F., Dubeau F., Gotman J. (2012). Patterns of altered functional connectivity in mesial temporal lobe epilepsy. Epilepsia.

[bb0270] Power J.D., Barnes K.A., Snyder A.Z., Schlaggar B.L., Petersen S.E. (2012). Spurious but systematic correlations in functional connectivity MRI networks arise from subject motion. NeuroImage.

[bb0275] Raffelt D., Tournier J.D., Rose S., Ridgway G.R., Henderson R., Crozier S., Salvado O., Connelly A. (2012). Apparent fibre density: a novel measure for the analysis of diffusion-weighted magnetic resonance images. NeuroImage.

[bb0280] Realmuto S., Zummo L., Cerami C., Agro L., Dodich A., Canessa N., Zizzo A., Fierro B., Daniele O. (2015). Social cognition dysfunctions in patients with epilepsy: evidence from patients with temporal lobe and idiopathic generalized epilepsies. Epilepsy Behav..

[bb0285] Ridley B.G.Y., Rousseau C., Wirsich J., Le Troter A., Soulier E., Confort-Gouny S., Bartolomei F., Ranjeva J.-P., Achard S., Guye M. (2015). Nodal approach reveals differential impact of lateralized focal epilepsies on hub reorganization. NeuroImage.

[bb0290] Robinson P.A., Sarkar S., Pandejee G.M., Henderson J.A. (2014). Determination of effective brain connectivity from functional connectivity with application to resting state connectivities. Phys. Rev. E Stat. Nonlinear Soft Matter Phys..

[bb0295] Rubinov M., Sporns O. (2010). Complex network measures of brain connectivity: uses and interpretations. NeuroImage.

[bb0300] Sled J.G., Zijdenbos A.P., Evans A.C. (1998). A nonparametric method for automatic correction of intensity nonuniformity in MRI data. IEEE Trans. Med. Imaging.

[bb0305] Smith S.M., Nichols T.E. (2009). Threshold-free cluster enhancement: addressing problems of smoothing, threshold dependence and localisation in cluster inference. NeuroImage.

[bb0310] Smith S.M., Jenkinson M., Woolrich M.W., Beckmann C.F., Behrens T.E., Johansen-Berg H., Bannister P.R., De Luca M., Drobnjak I., Flitney D.E. (2004). Advances in functional and structural MR image analysis and implementation as FSL. NeuroImage.

[bb0315] Spencer S.S. (2002). Neural networks in human epilepsy: evidence of and implications for treatment. Epilepsia.

[bb0320] Sporns O. (2013). Structure and function of complex brain networks. Dialogues Clin. Neurosci..

[bb0325] Sporns O., Tononi G., Kotter R. (2005). The human connectome: a structural description of the human brain. PLoS Comput. Biol..

[bb0330] Su L., An J., Ma Q., Qiu S., Hu D. (2015). Influence of resting-state network on lateralization of functional connectivity in mesial temporal lobe epilepsy. AJNR Am. J. Neuroradiol..

[bb0335] Tagliazucchi E., Laufs H. (2015). Multimodal imaging of dynamic functional connectivity. Front. Neurol..

[bb0340] Thom M., Holton J.L., D'Arrigo C., Griffin B., Beckett A., Sisodiya S., Alexiou D., Sander J.W. (2000). Microdysgenesis with abnormal cortical myelinated fibres in temporal lobe epilepsy: a histopathological study with calbindin D-28-K immunohistochemistry. Neuropathol. Appl. Neurobiol..

[bb0345] Thom M., Sisodiya S., Harkness W., Scaravilli F. (2001). Microdysgenesis in temporal lobe epilepsy. A quantitative and immunohistochemical study of white matter neurones. Brain.

[bb0350] Tournier J.D., Yeh C.H., Calamante F., Cho K.H., Connelly A., Lin C.P. (2008). Resolving crossing fibres using constrained spherical deconvolution: validation using diffusion-weighted imaging phantom data. NeuroImage.

[bb0355] Tournier J., Calamante F., Connelly A. (2010). Improved probabilistic streamlines tractography by 2nd order integration over fibre orientation distributions. Proc. 18th Annual Meeting of the Intl. Soc. Mag. Reson. Med.(ISMRM).

[bb0360] Tournier J.D., Calamante F., Connelly A. (2012). MRtrix: diffusion tractography in crossing fiber regions. Int. J. Imaging Syst. Technol..

[bb0365] Tzourio-Mazoyer N., Landeau B., Papathanassiou D., Crivello F., Etard O., Delcroix N., Mazoyer B., Joliot M. (2002). Automated anatomical labeling of activations in SPM using a macroscopic anatomical parcellation of the MNI MRI single-subject brain. NeuroImage.

[bb0370] Vaessen M.J., Jansen J.F., Vlooswijk M.C., Hofman P.A., Majoie H.J., Aldenkamp A.P., Backes W.H. (2012). White matter network abnormalities are associated with cognitive decline in chronic epilepsy. Cereb. Cortex.

[bb0375] Vaessen M.J., Jansen J.F., Braakman H.M., Hofman P.A., De Louw A., Aldenkamp A.P., Backes W.H. (2014). Functional and structural network impairment in childhood frontal lobe epilepsy. PLoS One.

[bb0380] van den Heuvel M.P., Sporns O. (2011). Rich-club organization of the human connectome. J. Neurosci..

[bb0385] van den Heuvel M.P., Sporns O., Collin G., Scheewe T., Mandl R.C., Cahn W., Goni J., Hulshoff Pol H.E., Kahn R.S. (2013). Abnormal rich club organization and functional brain dynamics in schizophrenia. JAMA Psychiatry.

[bb0390] Voets N.L., Beckmann C.F., Cole D.M., Hong S., Bernasconi A., Bernasconi N. (2012). Structural substrates for resting network disruption in temporal lobe epilepsy. Brain.

[bb0395] Zalesky A., Fornito A., Bullmore E.T. (2010). Network-based statistic: identifying differences in brain networks. NeuroImage.

[bb0400] Zalesky A., Fornito A., Harding I.H., Cocchi L., Yucel M., Pantelis C., Bullmore E.T. (2010). Whole-brain anatomical networks: does the choice of nodes matter?. NeuroImage.

[bb0405] Zalesky A., Fornito A., Cocchi L., Gollo L.L., Breakspear M. (2014). Time-resolved resting-state brain networks. Proc. Natl. Acad. Sci. U. S. A..

[bb0410] Zhang Z., Liao W., Chen H., Mantini D., Ding J.R., Xu Q., Wang Z., Yuan C., Chen G., Jiao Q., Lu G. (2011). Altered functional-structural coupling of large-scale brain networks in idiopathic generalized epilepsy. Brain.

