# Supplementary material for: Whole-brain analytic measures of network communication reveal increased structure-function correlation in right temporal lobe epilepsy
Source: Neuroimage Clin. 2016 May 19;11:707–18. doi: 10.1016/j.nicl.2016.05.010 (PMC4909094; doi:10.1016/j.nicl.2016.05.010)
Supplement: Supplementary material 1 — Right temporal lobe patients characteristics and average connectivity metric statistics. [file mmc1.docx]

**Supplementary Information 1 | Right temporal lobe patients characteristics and average connectivity metric statistics**

**Table 1.** epilepsy characteristics of rTLE patients, Abbreviations used - FCD: Focal Cortical Dysplasia; HS: Hippocampal Sclerosis; GG: ganglioglioma; MR: Magnetic resonance

| **#patient** | **handedness** | **age** | **onset (years)** | **frequency** | **duration (years)** | **MR imaging** |
| --- | --- | --- | --- | --- | --- | --- |
| **1** | Right | 38 | 7 | 2/month | 31 | MR Normal |
| **2** | Right | 29 | 17 | 1/6 month | 12 | right HS |
| **3** | Right | 51 | 23 | 2/day | 28 | right temporal atrophy |
| **4** | Left | 25 | 0 | 2-3/month | 25 | Right temporal FCD |
| **5** | Right | 21 | 8 | 30-90/month | 13 | right temporal GG |
| **6** | Right | 36 | 10 | 3/month | 26 | MR Normal |
| **7** | Right | 31 | 28 | 3-4/month | 3 | FLAIR hypersignal right temporal pole |

**Table 2.** Mean value of all used metric and corresponding p-value for Wilcoxon rank-sum test between controls and rTLE patients (*) p<0.008 Bonferroni corrected

|  | **Mean Controls** | **Mean rTLE** | **P controls vs. rTLE** |
| --- | --- | --- | --- |
| **Streamline count (log normalized)** | 0.106 | 0.101 | 0.178 |
| **FCA (normalized)** | 0.123 | 0.112 | 0.057 |
| **Euclidian Distance (normalized)** | 0.482 | 0.480 | 0.428 |
| **Weighted Path length** | 43.041 | 40.011 | 0.526 |
| **Path transitivity** | 0.785 | 0.777 | 0.428 |
| **SI (log)** | 1.195 | 1.210 | 0.007 (*) |
