# Supplementary material for: Whole-brain analytic measures of network communication reveal increased structure-function correlation in right temporal lobe epilepsy
Source: Neuroimage Clin. 2016 May 19;11:707–18. doi: 10.1016/j.nicl.2016.05.010 (PMC4909094; doi:10.1016/j.nicl.2016.05.010)
Supplement: Supplementary material 2 — Parcellation Information. [file mmc2.docx]

**Supplementary Information 2 | Parcellation Information**

**SI Table 1.** Anatomical information for each parcellation region (Zalesky et al., 2010)

| Node ID | X-Centroid | Y-Centroid | Z-Centroid | AAL match | Lobe |
| --- | --- | --- | --- | --- | --- |
| 1 | -20 | -85 | 32 | Occipital Sup L.1 | Left Occipital |
| 2 | -48 | -16 | 45 | Postcentral L.1 | Left Frontal |
| 3 | -35 | -33 | 61 | Postcentral L.2 | Left Parietal |
| 4 | -57 | -13 | 23 | Postcentral L.3 | Left Frontal |
| 5 | -17 | 36 | 26 | Frontal Sup Medial L.1 | Left PreFrontal |
| 6 | -10 | -61 | 12 | Calcarine L.1 | Left Limbic |
| 7 | -39 | -32 | 48 | Postcentral L.4 | Left Parietal |
| 8 | -40 | 7 | 22 | Frontal Inf Oper L.1 | Left PreFrontal |
| 9 | -61 | -32 | -9 | Temporal Mid L.1 | Left Temporal |
| 10 | -53 | 1 | 34 | Precentral L.1 | Left Frontal |
| 11 | -56 | -24 | 27 | SupraMarginal L.1 | Left Parietal |
| 12 | -49 | 9 | 13 | Frontal Inf Oper L.2 | Left PreFrontal |
| 13 | -8 | -81 | 9 | Calcarine L.2 | Left Occipital |
| 14 | -29 | 53 | -8 | Frontal Mid Orb L.1 | Left PreFrontal |
| 15 | -50 | -56 | 27 | Angular L.1 | Left Parietal |
| 16 | -47 | -31 | 7 | Temporal Sup L.1 | Left Temporal |
| 17 | -27 | -88 | -13 | Occipital Inf L.1 | Left Occipital |
| 18 | -55 | -9 | -10 | Temporal Mid L.2 | Left Temporal |
| 19 | -37 | -80 | -5 | Occipital Mid L.1 | Left Occipital |
| 20 | -21 | -64 | 52 | Parietal Sup L.1 | Left Parietal |
| 21 | -49 | 16 | -18 | Temporal Pole Sup L.1 | Left Temporal |
| 22 | -57 | 5 | 19 | Precentral L.2 | Left PreFrontal |
| 23 | -33 | 43 | 29 | Frontal Mid L.1 | Left PreFrontal |
| 24 | -45 | 18 | -2 | Frontal Inf Tri L.1 | Left PreFrontal |
| 25 | -36 | -79 | 16 | Occipital Mid L.2 | Left Temporal |
| 26 | -21 | -8 | 0 | Putamen L.1 | Left Sub-Cortex |
| 27 | -34 | -74 | 26 | Occipital Mid L.3 | Left Parietal |
| 28 | -17 | 35 | 47 | Frontal Sup L.1 | Left PreFrontal |
| 29 | -11 | -26 | 35 | Cingulum Mid L.1 | Left Limbic |

| Node ID | X-Centroid | Y-Centroid | Z-Centroid | AAL match | Lobe |
| --- | --- | --- | --- | --- | --- |
| 30 | -10 | -74 | 19 | Cuneus L.1 | Left Occipital |
| 31 | -17 | 24 | 53 | Frontal Sup L.2 | Left PreFrontal |
| 32 | -10 | -82 | -8 | Lingual L.1 | Left Occipital |
| 33 | -26 | -7 | 9 | Insula L.1 | Left Sub-Cortex |
| 34 | -45 | -71 | 11 | Occipital Mid L.4 | Left Temporal |
| 35 | -26 | -70 | 41 | Parietal Sup L.2 | Left Parietal |
| 36 | -28 | -35 | 40 | Parietal Inf L.1 | Left Parietal |
| 37 | -12 | -21 | 66 | Paracentral Lobule L.1 | Left Frontal |
| 38 | -16 | -86 | 15 | Occipital Sup L.2 | Left Occipital |
| 39 | -12 | -17 | 40 | Cingulum Mid L.2 | Left Limbic |
| 40 | -35 | -13 | -32 | Temporal Inf L.1 | Left Temporal |
| 41 | -24 | -12 | 67 | Precentral L.3 | Left Frontal |
| 42 | -23 | -72 | 27 | Occipital Sup L.3 | Left Parietal |
| 43 | -50 | 16 | 20 | Frontal Inf Tri L.2 | Left PreFrontal |
| 44 | -34 | 35 | -15 | Frontal Inf Orb L.1 | Left PreFrontal |
| 45 | -51 | -14 | 29 | Postcentral L.5 | Left Frontal |
| 46 | -38 | -80 | -13 | Occipital Inf L.2 | Left Occipital |
| 47 | -11 | -85 | 27 | Cuneus L.2 | Left Occipital |
| 48 | -55 | -6 | -2 | Temporal Sup L.2 | Left Temporal |
| 49 | -43 | -66 | 0 | Occipital Mid L.5 | Left Temporal |
| 50 | -37 | -21 | 56 | Precentral L.4 | Left Frontal |
| 51 | -54 | -56 | 16 | Temporal Mid L.3 | Left Temporal |
| 52 | -38 | -51 | 45 | Parietal Inf L.2 | Left Parietal |
| 53 | -29 | -78 | 35 | Occipital Mid L.6 | Left Parietal |
| 54 | -34 | 31 | 22 | Frontal Mid L.2 | Left PreFrontal |
| 55 | -37 | -1 | 56 | Precentral L.5 | Left Frontal |
| 56 | -50 | -16 | -17 | Temporal Mid L.4 | Left Temporal |
| 57 | -18 | -54 | -6 | Lingual L.2 | Left Limbic |
| 58 | -11 | 1 | 35 | Cingulum Mid L.3 | Left Limbic |
| 59 | -34 | -62 | 29 | Occipital Mid L.7 | Left Parietal |
| 60 | -45 | 1 | -37 | Temporal Inf L.2 | Left Temporal |

| Node ID | X-Centroid | Y-Centroid | Z-Centroid | AAL match | Lobe |
| --- | --- | --- | --- | --- | --- |
| 61 | -33 | -9 | -3 | Putamen L.2 | Left Sub-Cortex |
| 62 | -26 | 10 | 50 | Frontal Mid L.3 | Left Frontal |
| 63 | -40 | -72 | 34 | Angular L.2 | Left Parietal |
| 64 | -29 | 0 | -40 | Fusiform L.1 | Left Temporal |
| 65 | -19 | -78 | 21 | Occipital Sup L.4 | Left Occipital |
| 66 | -12 | -45 | 64 | Precuneus L.1 | Left Parietal |
| 67 | -12 | -62 | 42 | Precuneus L.2 | Left Parietal |
| 68 | -50 | -44 | 34 | Parietal Inf L.3 | Left Parietal |
| 69 | -49 | -48 | -21 | Temporal Inf L.3 | Left Temporal |
| 70 | -45 | -63 | 33 | Angular L.3 | Left Parietal |
| 71 | -57 | -8 | 14 | Postcentral L.6 | Left Frontal |
| 72 | -36 | -51 | -14 | Fusiform L.2 | Left Temporal |
| 73 | -8 | 26 | -5 | Cingulum Ant L.1 | Left Limbic |
| 74 | -31 | -68 | -14 | Fusiform L.3 | Left Occipital |
| 75 | -44 | 22 | 23 | Frontal Inf Tri L.3 | Left PreFrontal |
| 76 | -42 | 40 | -10 | Frontal Inf Orb L.2 | Left PreFrontal |
| 77 | -17 | -60 | 32 | Precuneus L.3 | Left Parietal |
| 78 | -23 | 49 | -11 | Frontal Mid Orb L.2 | Left PreFrontal |
| 79 | -21 | -57 | 61 | Parietal Sup L.3 | Left Parietal |
| 80 | -15 | -40 | 44 | Cingulum Mid L.4 | Left Parietal |
| 81 | -52 | -22 | -28 | Temporal Inf L.4 | Left Temporal |
| 82 | -8 | -75 | 34 | Cuneus L.3 | Left Parietal |
| 83 | -8 | -11 | 62 | Supp Motor Area L.1 | Left Frontal |
| 84 | -15 | 18 | -18 | Rectus L.1 | Left PreFrontal |
| 85 | -15 | 10 | -10 | Putamen L.3 | Left Sub-Cortex |
| 86 | -11 | 35 | -9 | Rectus L.2 | Left PreFrontal |
| 87 | -18 | 16 | 27 | Cingulum Ant L.2 | Left Limbic |
| 88 | -49 | -3 | -29 | Temporal Mid L.5 | Left Temporal |
| 89 | -52 | 4 | -20 | Temporal Mid L.6 | Left Temporal |
| 90 | -24 | -19 | -11 | Hippocampus L.1 | Left Limbic |
| 91 | -9 | -53 | 46 | Precuneus L.4 | Left Parietal |

| Node ID | X-Centroid | Y-Centroid | Z-Centroid | AAL match | Lobe |
| --- | --- | --- | --- | --- | --- |
| 92 | -14 | -21 | 9 | Thalamus L.1 | Left Sub-Cortex |
| 93 | -30 | 43 | 12 | Frontal Mid L.4 | Left PreFrontal |
| 94 | -26 | -25 | -21 | ParaHippocampal L.1 | Left Limbic |
| 95 | -39 | 4 | -9 | Insula L.2 | Left Frontal-Temporal |
| 96 | -26 | -6 | -29 | ParaHippocampal L.2 | Left Limbic |
| 97 | -14 | 43 | 6 | Frontal Sup Medial L.2 | Left PreFrontal |
| 98 | -30 | -15 | 59 | Precentral L.6 | Left Frontal |
| 99 | -31 | 5 | 54 | Frontal Mid L.5 | Left Frontal |
| 100 | -25 | -50 | 64 | Parietal Sup L.4 | Left Parietal |
| 101 | -30 | -34 | 52 | Postcentral L.7 | Left Parietal |
| 102 | -36 | -22 | 10 | Heschl L.1 | Left Frontal-Temporal |
| 103 | -8 | 49 | -4 | Frontal Med Orb L.1 | Left PreFrontal |
| 104 | -11 | 1 | 63 | Supp Motor Area L.2 | Left Frontal |
| 105 | -8 | 35 | 13 | Cingulum Ant L.3 | Left Limbic |
| 106 | -29 | 10 | 0 | Putamen L.4 | Left Sub-Cortex |
| 107 | -24 | 30 | 36 | Frontal Mid L.6 | Left PreFrontal |
| 108 | -20 | 0 | -15 | Amygdala L.1 | Left Limbic |
| 109 | -42 | -54 | 37 | Angular L.4 | Left Parietal |
| 110 | -23 | 5 | -27 | Temporal Pole Sup L.2 | Left Limbic |
| 111 | -39 | -20 | -6 | Temporal Sup L.3 | Left Frontal-Temporal |
| 112 | -11 | -96 | -1 | Calcarine L.3 | Left Occipital |
| 113 | -8 | 1 | 52 | Supp Motor Area L.3 | Left Frontal |
| 114 | -11 | 8 | 51 | Supp Motor Area L.4 | Left Frontal |
| 115 | -17 | -13 | 62 | Paracentral Lobule L.2 | Left Frontal |
| 116 | -19 | -75 | 45 | Parietal Sup L.5 | Left Parietal |
| 117 | -14 | 4 | 15 | Caudate L.1 | Left Sub-Cortex |
| 118 | -57 | -34 | -17 | Temporal Mid L.7 | Left Temporal |
| 119 | -55 | -50 | -4 | Temporal Mid L.8 | Left Temporal |
| 120 | -17 | -88 | -14 | Lingual L.3 | Left Occipital |
| 121 | -47 | 10 | 37 | Precentral L.7 | Left PreFrontal |
| 122 | -42 | -7 | 0 | Insula L.3 | Left Frontal-Temporal |

| Node ID | X-Centroid | Y-Centroid | Z-Centroid | AAL match | Lobe |
| --- | --- | --- | --- | --- | --- |
| 123 | -13 | -95 | -10 | Calcarine L.4 | Left Occipital |
| 124 | -26 | 55 | 3 | Frontal Sup L.3 | Left PreFrontal |
| 125 | -9 | -72 | 44 | Precuneus L.5 | Left Parietal |
| 126 | -48 | -53 | 42 | Parietal Inf L.4 | Left Parietal |
| 127 | -61 | -49 | -13 | Temporal Inf L.5 | Left Temporal |
| 128 | -32 | -5 | -19 | Amygdala L.2 | Left Limbic |
| 129 | -9 | 34 | 43 | Frontal Sup Medial L.3 | Left PreFrontal |
| 130 | -57 | -45 | -22 | Temporal Inf L.6 | Left Temporal |
| 131 | -8 | 42 | 23 | Frontal Sup Medial L.4 | Left PreFrontal |
| 132 | -12 | 57 | -13 | Frontal Sup Orb L.1 | Left PreFrontal |
| 133 | -15 | -38 | 59 | Precuneus L.6 | Left Frontal |
| 134 | -41 | 14 | -34 | Temporal Pole Mid L.1 | Left Temporal |
| 135 | -51 | 24 | 5 | Frontal Inf Tri L.4 | Left PreFrontal |
| 136 | -31 | 19 | -31 | Temporal Pole Sup L.3 | Left Temporal |
| 137 | -47 | -30 | -23 | Temporal Inf L.7 | Left Temporal |
| 138 | -9 | -44 | 26 | Cingulum Post L.1 | Left Limbic |
| 139 | -52 | -33 | 23 | SupraMarginal L.2 | Left Parietal |
| 140 | -47 | -25 | 42 | Postcentral L.8 | Left Parietal |
| 141 | -42 | -10 | 12 | Rolandic Oper L.1 | Left Frontal-Temporal |
| 142 | -34 | 50 | 4 | Frontal Mid L.7 | Left PreFrontal |
| 143 | -45 | -71 | -13 | Occipital Inf L.3 | Left Occipital |
| 144 | -17 | 37 | 38 | Frontal Sup L.4 | Left PreFrontal |
| 145 | -49 | -57 | -14 | Temporal Inf L.8 | Left Temporal |
| 146 | -18 | 10 | 59 | Frontal Sup L.5 | Left Frontal |
| 147 | -7 | -90 | 5 | Calcarine L.5 | Left Occipital |
| 148 | -25 | -94 | 7 | Occipital Mid L.8 | Left Occipital |
| 149 | -60 | -35 | 18 | Temporal Sup L.4 | Left Temporal |
| 150 | -40 | -23 | -23 | Temporal Inf L.9 | Left Temporal |
| 151 | -9 | 26 | -20 | Rectus L.3 | Left PreFrontal |
| 152 | -24 | 40 | -17 | Frontal Mid Orb L.3 | Left PreFrontal |
| 153 | -58 | -17 | -24 | Temporal Inf L.10 | Left Temporal |

| Node ID | X-Centroid | Y-Centroid | Z-Centroid | AAL match | Lobe |
| --- | --- | --- | --- | --- | --- |
| 154 | -37 | 23 | -1 | Frontal Inf Tri L.5 | Left PreFrontal |
| 155 | -43 | -76 | 22 | Occipital Mid L.9 | Left Temporal |
| 156 | -21 | -64 | -4 | Lingual L.4 | Left Occipital |
| 157 | -31 | -54 | 54 | Parietal Sup L.6 | Left Parietal |
| 158 | -15 | -55 | 23 | Precuneus L.7 | Left Parietal |
| 159 | -11 | 29 | 22 | Cingulum Ant L.4 | Left Limbic |
| 160 | -15 | 55 | -5 | Frontal Med Orb L.2 | Left PreFrontal |
| 161 | -8 | 56 | 18 | Frontal Sup Medial L.5 | Left PreFrontal |
| 162 | -20 | 48 | 25 | Frontal Sup L.6 | Left PreFrontal |
| 163 | -12 | -72 | 6 | Calcarine L.6 | Left Occipital |
| 164 | -55 | -37 | 37 | Parietal Inf L.5 | Left Parietal |
| 165 | -49 | -10 | 36 | Postcentral L.9 | Left Frontal |
| 166 | -45 | 34 | -2 | Frontal Inf Tri L.6 | Left PreFrontal |
| 167 | -44 | 1 | 32 | Precentral L.8 | Left Frontal |
| 168 | -34 | 6 | -31 | Temporal Pole Sup L.4 | Left Temporal |
| 169 | -42 | 28 | 12 | Frontal Inf Tri L.7 | Left PreFrontal |
| 170 | -31 | -83 | 2 | Occipital Mid L.10 | Left Occipital |
| 171 | -32 | 20 | 46 | Frontal Mid L.8 | Left PreFrontal |
| 172 | -57 | -21 | 8 | Temporal Sup L.5 | Left Temporal |
| 173 | -30 | -21 | -27 | Fusiform L.4 | Left Limbic |
| 174 | -27 | -59 | 44 | Parietal Sup L.7 | Left Parietal |
| 175 | -33 | -33 | -12 | Hippocampus L.2 | Left Limbic |
| 176 | -10 | 15 | 34 | Cingulum Mid L.5 | Left Limbic |
| 177 | -22 | 24 | -22 | Frontal Inf Orb L.3 | Left PreFrontal |
| 178 | -54 | 4 | -1 | Rolandic Oper L.2 | Left Frontal-Temporal |
| 179 | -9 | -37 | 36 | Cingulum Mid L.6 | Left Limbic |
| 180 | -28 | -86 | 13 | Occipital Mid L.11 | Left Occipital |
| 181 | -29 | -5 | 50 | Precentral L.9 | Left Frontal |
| 182 | -43 | 8 | -26 | Temporal Pole Mid L.2 | Left Temporal |
| 183 | -35 | -74 | -1 | Occipital Mid L.12 | Left Occipital |
| 184 | -35 | 27 | 37 | Frontal Mid L.9 | Left PreFrontal |

| Node ID | X-Centroid | Y-Centroid | Z-Centroid | AAL match | Lobe |
| --- | --- | --- | --- | --- | --- |
| 185 | -14 | -94 | 13 | Occipital Sup L.5 | Left Occipital |
| 186 | -16 | 14 | 8 | Caudate L.2 | Left Sub-Cortex |
| 187 | -24 | 18 | 50 | Frontal Mid L.10 | Left PreFrontal |
| 188 | -34 | -57 | -17 | Fusiform L.5 | Left Occipital |
| 189 | -26 | 46 | 23 | Frontal Mid L.11 | Left PreFrontal |
| 190 | -12 | 60 | 9 | Frontal Sup Medial L.6 | Left PreFrontal |
| 191 | -30 | 13 | -22 | Temporal Pole Sup L.5 | Left Temporal |
| 192 | -15 | -30 | 0 | Thalamus L.2 | Left Limbic |
| 193 | -43 | -38 | 55 | Postcentral L.10 | Left Parietal |
| 194 | -53 | -56 | 7 | Temporal Mid L.9 | Left Temporal |
| 195 | -42 | -14 | 50 | Precentral L.10 | Left Frontal |
| 196 | -22 | 11 | -5 | Putamen L.5 | Left Sub-Cortex |
| 197 | -15 | 45 | 34 | Frontal Sup L.7 | Left PreFrontal |
| 198 | -26 | 25 | -13 | Frontal Inf Orb L.4 | Left PreFrontal |
| 199 | -7 | -59 | 24 | Precuneus L.8 | Left Parietal |
| 200 | -15 | -35 | 69 | Paracentral Lobule L.3 | Left Parietal |
| 201 | -49 | 16 | 31 | Frontal Inf Oper L.3 | Left PreFrontal |
| 202 | -13 | -60 | 59 | Precuneus L.9 | Left Parietal |
| 203 | -44 | 0 | 48 | Precentral L.11 | Left Frontal |
| 204 | -35 | 51 | 15 | Frontal Mid L.12 | Left PreFrontal |
| 205 | -7 | -34 | 50 | Paracentral Lobule L.4 | Left Frontal |
| 206 | -60 | -21 | -15 | Temporal Mid L.10 | Left Temporal |
| 207 | -20 | -49 | 0 | Lingual L.5 | Left Limbic |
| 208 | -42 | 30 | 32 | Frontal Mid L.13 | Left PreFrontal |
| 209 | -40 | -53 | 13 | Temporal Mid L.11 | Left Temporal |
| 210 | -51 | -44 | 22 | SupraMarginal L.3 | Left Parietal |
| 211 | -57 | -43 | 10 | Temporal Sup L.6 | Left Temporal |
| 212 | -43 | 15 | -13 | Temporal Pole Sup L.6 | Left PreFrontal |
| 213 | -49 | -3 | 12 | Rolandic Oper L.3 | Left Frontal |
| 214 | -23 | -56 | 17 | Angular L.5 | Left Limbic |
| 215 | -44 | 42 | 13 | Frontal Mid L.14 | Left PreFrontal |

| Node ID | X-Centroid | Y-Centroid | Z-Centroid | AAL match | Lobe |
| --- | --- | --- | --- | --- | --- |
| 216 | -41 | -29 | 14 | Rolandic Oper L.4 | Left Frontal-Temporal |
| 217 | -49 | -20 | 2 | Temporal Sup L.7 | Left Temporal |
| 218 | -35 | -63 | 51 | Parietal Sup L.8 | Left Parietal |
| 219 | -52 | -30 | 15 | Temporal Sup L.8 | Left Parietal |
| 220 | -17 | -47 | 49 | Precuneus L.10 | Left Parietal |
| 221 | -51 | -62 | -6 | Temporal Mid L.12 | Left Temporal |
| 222 | -23 | -23 | 70 | Precentral L.12 | Left Frontal |
| 223 | -27 | -4 | 21 | Precentral L.13 | Left Sub-Cortex |
| 224 | -36 | 19 | 32 | Frontal Mid L.15 | Left PreFrontal |
| 225 | -20 | -44 | 71 | Parietal Sup L.9 | Left Parietal |
| 226 | -36 | 4 | 43 | Precentral L.14 | Left Frontal |
| 227 | -22 | -30 | 62 | Postcentral L.11 | Left Frontal |
| 228 | -46 | 4 | 5 | Frontal Inf Oper L.4 | Left Frontal-Temporal |
| 229 | -18 | -69 | 0 | Lingual L.6 | Left Occipital |
| 230 | -10 | 23 | 49 | Supp Motor Area L.5 | Left PreFrontal |
| 231 | -10 | 53 | 30 | Frontal Sup Medial L.7 | Left PreFrontal |
| 232 | -21 | -36 | 7 | Hippocampus L.3 | Left Limbic |
| 233 | -42 | -41 | -21 | Temporal Inf L.11 | Left Temporal |
| 234 | -58 | -35 | 2 | Temporal Mid L.13 | Left Temporal |
| 235 | -30 | -9 | 41 | Precentral L.15 | Left Frontal |
| 236 | -40 | 11 | 3 | Insula L.4 | Left Frontal-Temporal |
| 237 | -49 | -63 | 5 | Temporal Mid L.14 | Left Temporal |
| 238 | -29 | 22 | 5 | Frontal Inf Tri L.8 | Left PreFrontal |
| 239 | -44 | -30 | 35 | Parietal Inf L.6 | Left Parietal |
| 240 | -16 | -12 | 52 | Supp Motor Area L.6 | Left Frontal |
| 241 | -53 | -6 | -20 | Temporal Mid L.15 | Left Temporal |
| 242 | -9 | 45 | -15 | Rectus L.4 | Left PreFrontal |
| 243 | -49 | 29 | -6 | Frontal Inf Orb L.5 | Left PreFrontal |
| 244 | -7 | -10 | 36 | Cingulum Mid L.7 | Left Limbic |
| 245 | -31 | -92 | -3 | Occipital Mid L.13 | Left Occipital |
| 246 | -23 | -37 | -10 | ParaHippocampal L.3 | Left Limbic |

| Node ID | X-Centroid | Y-Centroid | Z-Centroid | AAL match | Lobe |
| --- | --- | --- | --- | --- | --- |
| 247 | -40 | -48 | 55 | Parietal Inf L.7 | Left Parietal |
| 248 | -50 | -34 | 46 | Parietal Inf L.8 | Left Parietal |
| 249 | -42 | 35 | 14 | Frontal Inf Tri L.9 | Left PreFrontal |
| 250 | -28 | -45 | -9 | Fusiform L.6 | Left Limbic |
| 251 | -42 | 44 | 1 | Frontal Mid L.16 | Left PreFrontal |
| 252 | -63 | -23 | -2 | Temporal Mid L.16 | Left Temporal |
| 253 | -21 | 60 | 10 | Frontal Sup L.8 | Left PreFrontal |
| 254 | -13 | 16 | 43 | Supp Motor Area L.7 | Left PreFrontal |
| 255 | -12 | -70 | -4 | Lingual L.7 | Left Occipital |
| 256 | -56 | -42 | -3 | Temporal Mid L.17 | Left Temporal |
| 257 | 19 | -85 | 33 | Occipital Sup R.1 | Right Occipital |
| 258 | 47 | -15 | 45 | Postcentral R.1 | Right Frontal |
| 259 | 34 | -32 | 62 | Postcentral R.2 | Right Parietal |
| 260 | 57 | -11 | 24 | Postcentral R.3 | Right Frontal |
| 261 | 16 | 36 | 26 | Frontal Sup R.1 | Right PreFrontal |
| 262 | 10 | -61 | 13 | Calcarine R.1 | Right Limbic |
| 263 | 37 | -32 | 48 | Postcentral R.4 | Right Parietal |
| 264 | 39 | 7 | 22 | Frontal Inf Oper R.1 | Right PreFrontal |
| 265 | 60 | -30 | -8 | Temporal Mid R.1 | Right Temporal |
| 266 | 53 | 2 | 35 | Precentral R.1 | Right Frontal |
| 267 | 55 | -24 | 28 | SupraMarginal R.1 | Right Parietal |
| 268 | 48 | 11 | 13 | Frontal Inf Oper R.2 | Right PreFrontal |
| 269 | 7 | -81 | 9 | Calcarine R.2 | Right Occipital |
| 270 | 28 | 53 | -8 | Frontal Mid Orb R.1 | Right PreFrontal |
| 271 | 49 | -54 | 28 | Angular R.1 | Right Parietal |
| 272 | 46 | -30 | 8 | Temporal Sup R.1 | Right Temporal |
| 273 | 27 | -88 | -12 | Occipital Inf R.1 | Right Occipital |
| 274 | 54 | -7 | -10 | Temporal Sup R.2 | Right Temporal |
| 275 | 36 | -79 | -5 | Occipital Inf R.2 | Right Occipital |
| 276 | 20 | -64 | 52 | Parietal Sup R.1 | Right Parietal |
| 277 | 48 | 17 | -17 | Temporal Pole Sup R.1 | Right Temporal |

| Node ID | X-Centroid | Y-Centroid | Z-Centroid | AAL match | Lobe |
| --- | --- | --- | --- | --- | --- |
| 278 | 56 | 7 | 19 | Precentral R.2 | Right PreFrontal |
| 279 | 31 | 43 | 29 | Frontal Mid R.1 | Right PreFrontal |
| 280 | 44 | 19 | -2 | Frontal Inf Orb R.1 | Right PreFrontal |
| 281 | 35 | -79 | 17 | Occipital Mid R.1 | Right Temporal |
| 282 | 20 | -8 | 1 | Putamen R.1 | Right Sub-Cortex |
| 283 | 33 | -73 | 27 | Occipital Mid R.2 | Right Parietal |
| 284 | 17 | 35 | 47 | Frontal Sup R.2 | Right PreFrontal |
| 285 | 10 | -26 | 35 | Cingulum Mid R.1 | Right Limbic |
| 286 | 10 | -74 | 19 | Cuneus R.1 | Right Occipital |
| 287 | 16 | 24 | 54 | Frontal Sup R.3 | Right PreFrontal |
| 288 | 10 | -82 | -8 | Lingual R.1 | Right Occipital |
| 289 | 24 | -7 | 9 | Insula R.1 | Right Sub-Cortex |
| 290 | 44 | -70 | 12 | Temporal Mid R.2 | Right Temporal |
| 291 | 25 | -69 | 41 | Occipital Sup R.2 | Right Parietal |
| 292 | 26 | -34 | 40 | Postcentral R.5 | Right Parietal |
| 293 | 11 | -21 | 66 | Precentral R.3 | Right Frontal |
| 294 | 16 | -86 | 14 | Occipital Sup R.3 | Right Occipital |
| 295 | 11 | -17 | 40 | Cingulum Mid R.2 | Right Limbic |
| 296 | 35 | -12 | -33 | Fusiform R.1 | Right Temporal |
| 297 | 23 | -11 | 67 | Precentral R.4 | Right Frontal |
| 298 | 23 | -72 | 28 | Occipital Sup R.4 | Right Parietal |
| 299 | 49 | 16 | 20 | Frontal Inf Tri R.1 | Right PreFrontal |
| 300 | 34 | 36 | -14 | Frontal Inf Orb R.2 | Right PreFrontal |
| 301 | 51 | -14 | 30 | Postcentral R.6 | Right Frontal |
| 302 | 38 | -79 | -12 | Occipital Inf R.3 | Right Occipital |
| 303 | 11 | -85 | 28 | Cuneus R.2 | Right Occipital |
| 304 | 55 | -5 | -2 | Temporal Sup R.3 | Right Temporal |
| 305 | 42 | -65 | 0 | Temporal Mid R.3 | Right Temporal |
| 306 | 37 | -20 | 57 | Precentral R.5 | Right Frontal |
| 307 | 53 | -56 | 17 | Temporal Mid R.4 | Right Temporal |
| 308 | 38 | -50 | 46 | Parietal Inf R.1 | Right Parietal |

| Node ID | X-Centroid | Y-Centroid | Z-Centroid | AAL match | Lobe |
| --- | --- | --- | --- | --- | --- |
| 309 | 28 | -78 | 35 | Occipital Mid R.3 | Right Parietal |
| 310 | 33 | 32 | 22 | Frontal Mid R.2 | Right PreFrontal |
| 311 | 36 | 0 | 56 | Frontal Mid R.3 | Right Frontal |
| 312 | 49 | -15 | -16 | Temporal Mid R.5 | Right Temporal |
| 313 | 17 | -53 | -6 | Lingual R.2 | Right Limbic |
| 314 | 10 | 2 | 35 | Cingulum Mid R.3 | Right Limbic |
| 315 | 33 | -61 | 30 | Angular R.2 | Right Parietal |
| 316 | 44 | 2 | -36 | Temporal Inf R.1 | Right Temporal |
| 317 | 32 | -7 | -1 | Putamen R.2 | Right Sub-Cortex |
| 318 | 25 | 11 | 50 | Frontal Mid R.4 | Right Frontal |
| 319 | 40 | -70 | 35 | Angular R.3 | Right Parietal |
| 320 | 28 | 1 | -40 | Fusiform R.2 | Right Temporal |
| 321 | 18 | -78 | 21 | Occipital Sup R.5 | Right Occipital |
| 322 | 11 | -44 | 64 | Postcentral R.7 | Right Parietal |
| 323 | 11 | -62 | 42 | Precuneus R.1 | Right Parietal |
| 324 | 49 | -43 | 34 | SupraMarginal R.2 | Right Parietal |
| 325 | 48 | -47 | -21 | Temporal Inf R.2 | Right Temporal |
| 326 | 44 | -61 | 33 | Angular R.4 | Right Parietal |
| 327 | 56 | -7 | 16 | Postcentral R.8 | Right Frontal |
| 328 | 36 | -51 | -14 | Fusiform R.3 | Right Temporal |
| 329 | 9 | 25 | -4 | Caudate R.1 | Right Limbic |
| 330 | 31 | -67 | -14 | Fusiform R.4 | Right Occipital |
| 331 | 43 | 24 | 25 | Frontal Inf Tri R.2 | Right PreFrontal |
| 332 | 42 | 41 | -9 | Frontal Inf Orb R.3 | Right PreFrontal |
| 333 | 16 | -60 | 32 | Precuneus R.2 | Right Parietal |
| 334 | 22 | 49 | -10 | Frontal Mid Orb R.2 | Right PreFrontal |
| 335 | 20 | -57 | 61 | Parietal Sup R.2 | Right Parietal |
| 336 | 15 | -40 | 44 | Precuneus R.3 | Right Parietal |
| 337 | 51 | -21 | -27 | Temporal Inf R.3 | Right Temporal |
| 338 | 8 | -74 | 34 | Cuneus R.3 | Right Parietal |
| 339 | 7 | -11 | 62 | Supp Motor Area R.1 | Right Frontal |

| Node ID | X-Centroid | Y-Centroid | Z-Centroid | AAL match | Lobe |
| --- | --- | --- | --- | --- | --- |
| 340 | 14 | 19 | -17 | Frontal Sup Orb R.1 | Right PreFrontal |
| 341 | 14 | 10 | -10 | Putamen R.3 | Right Sub-Cortex |
| 342 | 11 | 36 | -9 | Frontal Med Orb R.1 | Right PreFrontal |
| 343 | 16 | 16 | 27 | Cingulum Ant R.1 | Right Limbic |
| 344 | 49 | -1 | -29 | Temporal Mid R.6 | Right Temporal |
| 345 | 52 | 5 | -19 | Temporal Pole Mid R.1 | Right Temporal |
| 346 | 24 | -18 | -12 | Hippocampus R.1 | Right Limbic |
| 347 | 8 | -53 | 47 | Precuneus R.4 | Right Parietal |
| 348 | 14 | -21 | 10 | Thalamus R.1 | Right Sub-Cortex |
| 349 | 29 | 43 | 12 | Frontal Mid R.5 | Right PreFrontal |
| 350 | 26 | -25 | -20 | ParaHippocampal R.1 | Right Limbic |
| 351 | 39 | 4 | -8 | Insula R.2 | Right Frontal-Temporal |
| 352 | 26 | -6 | -28 | ParaHippocampal R.2 | Right Limbic |
| 353 | 13 | 45 | 7 | Frontal Sup Medial R.1 | Right PreFrontal |
| 354 | 29 | -14 | 59 | Precentral R.6 | Right Frontal |
| 355 | 30 | 5 | 54 | Frontal Mid R.6 | Right Frontal |
| 356 | 25 | -49 | 64 | Parietal Sup R.3 | Right Parietal |
| 357 | 29 | -34 | 52 | Postcentral R.9 | Right Parietal |
| 358 | 35 | -20 | 11 | Heschl R.1 | Right Frontal-Temporal |
| 359 | 8 | 49 | -3 | Frontal Med Orb R.2 | Right PreFrontal |
| 360 | 10 | 1 | 63 | Supp Motor Area R.2 | Right Frontal |
| 361 | 7 | 35 | 13 | Cingulum Ant R.2 | Right Limbic |
| 362 | 29 | 11 | 0 | Putamen R.4 | Right Sub-Cortex |
| 363 | 23 | 30 | 36 | Frontal Mid R.7 | Right PreFrontal |
| 364 | 19 | 1 | -15 | ParaHippocampal R.3 | Right Limbic |
| 365 | 41 | -53 | 38 | Angular R.5 | Right Parietal |
| 366 | 23 | 5 | -25 | ParaHippocampal R.4 | Right Limbic |
| 367 | 39 | -19 | -6 | Temporal Sup R.4 | Right Frontal-Temporal |
| 368 | 10 | -96 | -1 | Calcarine R.3 | Right Occipital |
| 369 | 7 | 1 | 52 | Supp Motor Area R.3 | Right Frontal |
| 370 | 10 | 8 | 51 | Supp Motor Area R.4 | Right Frontal |

| Node ID | X-Centroid | Y-Centroid | Z-Centroid | AAL match | Lobe |
| --- | --- | --- | --- | --- | --- |
| 371 | 16 | -13 | 62 | Frontal Sup R.4 | Right Frontal |
| 372 | 18 | -74 | 45 | Parietal Sup R.4 | Right Parietal |
| 373 | 14 | 4 | 15 | Caudate R.2 | Right Sub-Cortex |
| 374 | 57 | -33 | -16 | Temporal Inf R.4 | Right Temporal |
| 375 | 55 | -48 | -4 | Temporal Mid R.7 | Right Temporal |
| 376 | 17 | -88 | -14 | Lingual R.3 | Right Occipital |
| 377 | 47 | 11 | 36 | Precentral R.7 | Right PreFrontal |
| 378 | 41 | -6 | 1 | Insula R.3 | Right Frontal-Temporal |
| 379 | 13 | -95 | -10 | Lingual R.4 | Right Occipital |
| 380 | 24 | 55 | 3 | Frontal Sup R.5 | Right PreFrontal |
| 381 | 8 | -72 | 44 | Precuneus R.5 | Right Parietal |
| 382 | 48 | -52 | 43 | Parietal Inf R.2 | Right Parietal |
| 383 | 60 | -48 | -12 | Temporal Inf R.5 | Right Temporal |
| 384 | 31 | -4 | -18 | Amygdala R.1 | Right Limbic |
| 385 | 8 | 34 | 43 | Frontal Sup Medial R.2 | Right PreFrontal |
| 386 | 57 | -43 | -21 | Temporal Inf R.6 | Right Temporal |
| 387 | 7 | 42 | 23 | Cingulum Ant R.3 | Right PreFrontal |
| 388 | 11 | 57 | -13 | Frontal Sup Orb R.2 | Right PreFrontal |
| 389 | 14 | -38 | 59 | Postcentral R.10 | Right Frontal |
| 390 | 41 | 15 | -33 | Temporal Pole Mid R.2 | Right Temporal |
| 391 | 51 | 25 | 5 | Frontal Inf Tri R.3 | Right PreFrontal |
| 392 | 30 | 19 | -31 | Temporal Pole Mid R.3 | Right Temporal |
| 393 | 46 | -29 | -23 | Temporal Inf R.7 | Right Temporal |
| 394 | 9 | -44 | 26 | Cingulum Post R.1 | Right Limbic |
| 395 | 51 | -31 | 24 | SupraMarginal R.3 | Right Parietal |
| 396 | 46 | -25 | 44 | Postcentral R.11 | Right Parietal |
| 397 | 41 | -9 | 13 | Rolandic Oper R.1 | Right Frontal-Temporal |
| 398 | 33 | 51 | 4 | Frontal Mid R.8 | Right PreFrontal |
| 399 | 44 | -70 | -12 | Occipital Inf R.4 | Right Occipital |
| 400 | 15 | 37 | 38 | Frontal Sup R.6 | Right PreFrontal |
| 401 | 48 | -56 | -14 | Temporal Inf R.8 | Right Temporal |

| Node ID | X-Centroid | Y-Centroid | Z-Centroid | AAL match | Lobe |
| --- | --- | --- | --- | --- | --- |
| 402 | 17 | 10 | 59 | Frontal Sup R.7 | Right Frontal |
| 403 | 6 | -90 | 5 | Calcarine R.4 | Right Occipital |
| 404 | 24 | -94 | 8 | Occipital Mid R.4 | Right Occipital |
| 405 | 60 | -34 | 19 | Temporal Sup R.5 | Right Temporal |
| 406 | 39 | -22 | -22 | Fusiform R.5 | Right Temporal |
| 407 | 9 | 26 | -20 | Rectus R.1 | Right PreFrontal |
| 408 | 23 | 39 | -16 | Frontal Mid Orb R.3 | Right PreFrontal |
| 409 | 57 | -16 | -25 | Temporal Inf R.9 | Right Temporal |
| 410 | 37 | 24 | -1 | Insula R.4 | Right PreFrontal |
| 411 | 42 | -75 | 23 | Occipital Mid R.5 | Right Temporal |
| 412 | 21 | -63 | -4 | Lingual R.5 | Right Occipital |
| 413 | 30 | -53 | 54 | Parietal Sup R.5 | Right Parietal |
| 414 | 14 | -55 | 23 | Precuneus R.6 | Right Parietal |
| 415 | 10 | 29 | 22 | Cingulum Ant R.4 | Right Limbic |
| 416 | 14 | 55 | -5 | Frontal Sup Orb R.3 | Right PreFrontal |
| 417 | 8 | 57 | 19 | Frontal Sup Medial R.3 | Right PreFrontal |
| 418 | 19 | 49 | 25 | Frontal Mid R.9 | Right PreFrontal |
| 419 | 12 | -72 | 7 | Calcarine R.5 | Right Occipital |
| 420 | 54 | -36 | 38 | SupraMarginal R.4 | Right Parietal |
| 421 | 47 | -9 | 36 | Postcentral R.12 | Right Frontal |
| 422 | 45 | 35 | -1 | Frontal Inf Tri R.4 | Right PreFrontal |
| 423 | 43 | 2 | 33 | Precentral R.8 | Right Frontal |
| 424 | 33 | 6 | -30 | Temporal Pole Mid R.4 | Right Temporal |
| 425 | 41 | 29 | 12 | Frontal Inf Tri R.5 | Right PreFrontal |
| 426 | 30 | -82 | 2 | Occipital Mid R.6 | Right Occipital |
| 427 | 31 | 21 | 46 | Frontal Mid R.10 | Right PreFrontal |
| 428 | 56 | -19 | 9 | Temporal Sup R.6 | Right Temporal |
| 429 | 30 | -20 | -27 | Fusiform R.6 | Right Limbic |
| 430 | 26 | -58 | 45 | Angular R.6 | Right Parietal |
| 431 | 32 | -33 | -12 | Fusiform R.7 | Right Limbic |
| 432 | 9 | 15 | 35 | Cingulum Mid R.4 | Right Limbic |

| Node ID | X-Centroid | Y-Centroid | Z-Centroid | AAL match | Lobe |
| --- | --- | --- | --- | --- | --- |
| 433 | 21 | 25 | -22 | Frontal Inf Orb R.4 | Right PreFrontal |
| 434 | 54 | 4 | 0 | Temporal Pole Sup R.2 | Right Frontal-Temporal |
| 435 | 8 | -37 | 36 | Cingulum Mid R.5 | Right Limbic |
| 436 | 27 | -85 | 12 | Occipital Mid R.7 | Right Occipital |
| 437 | 28 | -4 | 50 | Precentral R.9 | Right Frontal |
| 438 | 43 | 9 | -26 | Temporal Pole Mid R.5 | Right Temporal |
| 439 | 34 | -73 | 0 | Occipital Mid R.8 | Right Occipital |
| 440 | 34 | 28 | 38 | Frontal Mid R.11 | Right PreFrontal |
| 441 | 13 | -94 | 13 | Occipital Sup R.6 | Right Occipital |
| 442 | 16 | 14 | 9 | Caudate R.3 | Right Sub-Cortex |
| 443 | 23 | 19 | 51 | Frontal Mid R.12 | Right PreFrontal |
| 444 | 33 | -56 | -16 | Fusiform R.8 | Right Occipital |
| 445 | 25 | 47 | 23 | Frontal Mid R.13 | Right PreFrontal |
| 446 | 11 | 60 | 9 | Frontal Sup Medial R.4 | Right PreFrontal |
| 447 | 30 | 14 | -23 | Temporal Pole Sup R.3 | Right Temporal |
| 448 | 14 | -30 | 0 | Thalamus R.2 | Right Limbic |
| 449 | 43 | -36 | 55 | Parietal Inf R.3 | Right Parietal |
| 450 | 52 | -54 | 7 | Temporal Mid R.8 | Right Temporal |
| 451 | 41 | -13 | 51 | Precentral R.10 | Right Frontal |
| 452 | 21 | 11 | -4 | Putamen R.5 | Right Sub-Cortex |
| 453 | 15 | 46 | 35 | Frontal Sup R.8 | Right PreFrontal |
| 454 | 26 | 25 | -13 | Frontal Inf Orb R.5 | Right PreFrontal |
| 455 | 7 | -59 | 24 | Precuneus R.7 | Right Parietal |
| 456 | 14 | -35 | 70 | Postcentral R.13 | Right Parietal |
| 457 | 48 | 17 | 30 | Frontal Inf Oper R.3 | Right PreFrontal |
| 458 | 12 | -60 | 58 | Parietal Sup R.6 | Right Parietal |
| 459 | 43 | 1 | 48 | Precentral R.11 | Right Frontal |
| 460 | 35 | 52 | 16 | Frontal Mid R.14 | Right PreFrontal |
| 461 | 6 | -33 | 50 | Paracentral Lobule R.1 | Right Frontal |
| 462 | 59 | -20 | -15 | Temporal Mid R.9 | Right Temporal |
| 463 | 18 | -48 | 1 | Lingual R.6 | Right Limbic |

| Node ID | X-Centroid | Y-Centroid | Z-Centroid | AAL match | Lobe |
| --- | --- | --- | --- | --- | --- |
| 464 | 41 | 31 | 33 | Frontal Mid R.15 | Right PreFrontal |
| 465 | 40 | -52 | 13 | Temporal Mid R.10 | Right Temporal |
| 466 | 51 | -44 | 23 | Temporal Sup R.7 | Right Parietal |
| 467 | 56 | -41 | 10 | Temporal Mid R.11 | Right Temporal |
| 468 | 42 | 16 | -13 | Temporal Pole Sup R.4 | Right PreFrontal |
| 469 | 48 | -2 | 14 | Rolandic Oper R.2 | Right Frontal |
| 470 | 23 | -56 | 18 | Precuneus R.8 | Right Limbic |
| 471 | 43 | 43 | 14 | Frontal Mid R.16 | Right PreFrontal |
| 472 | 40 | -28 | 14 | Temporal Sup R.8 | Right Frontal-Temporal |
| 473 | 48 | -19 | 3 | Temporal Sup R.9 | Right Temporal |
| 474 | 33 | -62 | 51 | Angular R.7 | Right Parietal |
| 475 | 52 | -28 | 16 | Temporal Sup R.10 | Right Parietal |
| 476 | 16 | -46 | 49 | Parietal Sup R.7 | Right Parietal |
| 477 | 50 | -61 | -6 | Temporal Inf R.10 | Right Temporal |
| 478 | 22 | -22 | 70 | Precentral R.12 | Right Frontal |
| 479 | 25 | -4 | 21 | Precentral R.13 | Right Sub-Cortex |
| 480 | 35 | 19 | 32 | Frontal Mid R.17 | Right PreFrontal |
| 481 | 19 | -44 | 71 | Postcentral R.14 | Right Parietal |
| 482 | 35 | 4 | 44 | Frontal Mid R.18 | Right Frontal |
| 483 | 21 | -29 | 63 | Postcentral R.15 | Right Frontal |
| 484 | 45 | 4 | 6 | Rolandic Oper R.3 | Right Frontal-Temporal |
| 485 | 17 | -69 | 0 | Lingual R.7 | Right Occipital |
| 486 | 9 | 23 | 49 | Supp Motor Area R.5 | Right PreFrontal |
| 487 | 9 | 53 | 30 | Frontal Sup Medial R.5 | Right PreFrontal |
| 488 | 20 | -37 | 7 | Calcarine R.6 | Right Limbic |
| 489 | 42 | -40 | -21 | Temporal Inf R.11 | Right Temporal |
| 490 | 57 | -34 | 3 | Temporal Mid R.12 | Right Temporal |
| 491 | 29 | -9 | 41 | Precentral R.14 | Right Frontal |
| 492 | 39 | 12 | 3 | Insula R.5 | Right Frontal-Temporal |
| 493 | 48 | -62 | 6 | Temporal Mid R.13 | Right Temporal |
| 494 | 28 | 23 | 5 | Insula R.6 | Right PreFrontal |

| Node ID | X-Centroid | Y-Centroid | Z-Centroid | AAL match | Lobe |
| --- | --- | --- | --- | --- | --- |
| 495 | 43 | -29 | 35 | SupraMarginal R.5 | Right Parietal |
| 496 | 15 | -12 | 52 | Frontal Sup R.9 | Right Frontal |
| 497 | 52 | -5 | -20 | Temporal Mid R.14 | Right Temporal |
| 498 | 9 | 44 | -14 | Rectus R.2 | Right PreFrontal |
| 499 | 48 | 30 | -6 | Frontal Inf Orb R.6 | Right PreFrontal |
| 500 | 6 | -10 | 36 | Cingulum Mid R.6 | Right Limbic |
| 501 | 31 | -91 | -3 | Occipital Inf R.5 | Right Occipital |
| 502 | 22 | -36 | -10 | ParaHippocampal R.5 | Right Limbic |
| 503 | 38 | -46 | 56 | Parietal Sup R.8 | Right Parietal |
| 504 | 50 | -33 | 46 | Parietal Inf R.4 | Right Parietal |
| 505 | 41 | 36 | 16 | Frontal Mid R.19 | Right PreFrontal |
| 506 | 28 | -45 | -9 | Fusiform R.9 | Right Limbic |
| 507 | 41 | 45 | 2 | Frontal Mid R.20 | Right PreFrontal |
| 508 | 62 | -21 | -1 | Temporal Sup R.11 | Right Temporal |
| 509 | 21 | 60 | 10 | Frontal Sup R.10 | Right PreFrontal |
| 510 | 12 | 16 | 43 | Frontal Sup R.11 | Right PreFrontal |
| 511 | 11 | -70 | -5 | Lingual R.8 | Right Occipital |
| 512 | 55 | -41 | -2 | Temporal Mid R.15 | Right Temporal |
