# Supplementary material for: Whole-brain analytic measures of network communication reveal increased structure-function correlation in right temporal lobe epilepsy
Source: Neuroimage Clin. 2016 May 19;11:707–18. doi: 10.1016/j.nicl.2016.05.010 (PMC4909094; doi:10.1016/j.nicl.2016.05.010)
Supplement: Supplementary material 3 — Patient data evaluation. [file mmc3.docx]

**Supplementary Information 3 | Patient data evaluation**


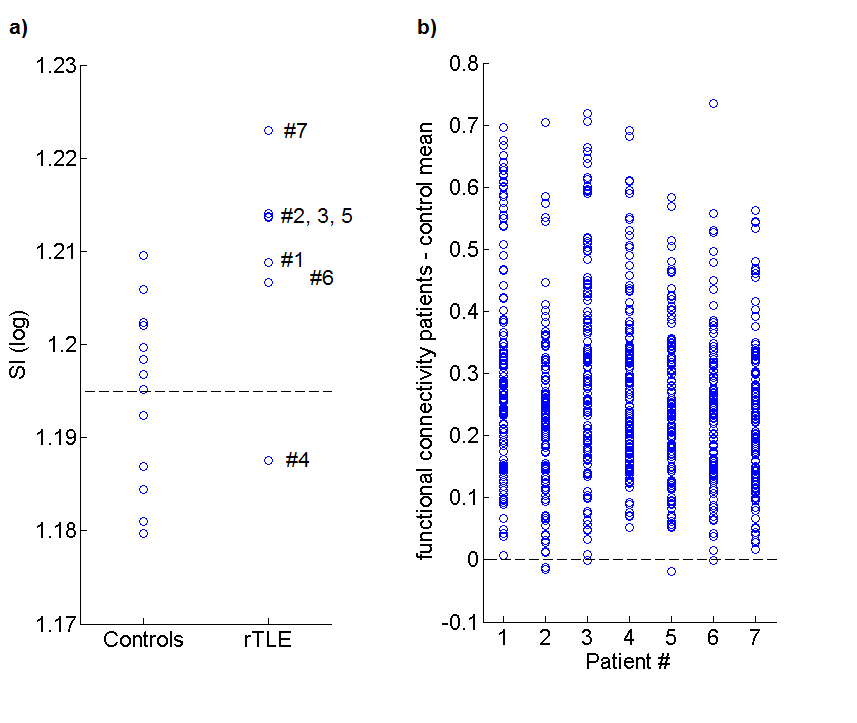


**SI Figure 1.** a) Distribution of mean search information (SI) of controls and rTLE patients, dotted line marks the controls’ mean, patients #1 and #6 are MR-normal with no visual lesions b) Distribution of the observed functional alteration subnetwork by using network based statistics (NBS). Each significant connection of the subnetwork is plotted by taking the patients correlation subtracted by the controls’ mean correlation (dotted line at 0 marks control mean). Over all patients, 5 individual regions were lower than the subjects’ mean for this connection. See SI1 for detailed patient characteristics.
