# Supplementary material for: Whole-brain analytic measures of network communication reveal increased structure-function correlation in right temporal lobe epilepsy
Source: Neuroimage Clin. 2016 May 19;11:707–18. doi: 10.1016/j.nicl.2016.05.010 (PMC4909094; doi:10.1016/j.nicl.2016.05.010)
Supplement: Supplementary material 4 — Network based statistics of functional connectivity. [file mmc4.docx]

**Supplementary Information 3 | Network based statistics of functional connectivity**

**SI Table 1.** rTLE patients > controls subnetwork identified by the NBS *p* < 0.05 (absolute functional connectivity)

| Source | Target | Source Label | Target Label |
| --- | --- | --- | --- |
| 5 | 144 | Frontal_Sup_Medial_L | Frontal_Sup_L |
| 5 | 163 | Frontal_Sup_Medial_L | Calcarine_L |
| 16 | 49 | Temporal_Sup_L | Occipital_Mid_L |
| 16 | 427 | Temporal_Sup_L | Frontal_Mid_R |
| 16 | 443 | Temporal_Sup_L | Frontal_Mid_R |
| 18 | 165 | Temporal_Mid_L | Postcentral_L |
| 18 | 195 | Temporal_Mid_L | Precentral_L |
| 18 | 205 | Temporal_Mid_L | Paracentral_Lobule_L |
| 18 | 258 | Temporal_Mid_L | Postcentral_R |
| 18 | 266 | Temporal_Mid_L | Precentral_R |
| 23 | 184 | Frontal_Mid_L | Frontal_Mid_L |
| 26 | 144 | Putamen_L | Frontal_Sup_L |
| 37 | 205 | Paracentral_Lobule_L | Paracentral_Lobule_L |
| 37 | 225 | Paracentral_Lobule_L | Parietal_Sup_L |
| 39 | 158 | Cingulum_Mid_L | Precuneus_L |
| 56 | 159 | Temporal_Mid_L | Cingulum_Ant_L |
| 76 | 89 | Frontal_Inf_Orb_L | Temporal_Mid_L |
| 76 | 241 | Frontal_Inf_Orb_L | Temporal_Mid_L |
| 87 | 224 | Cingulum_Ant_L | Frontal_Mid_L |
| 87 | 231 | Cingulum_Ant_L | Frontal_Sup_Medial_L |
| 90 | 255 | Hippocampus_L | Lingual_L |
| 90 | 427 | Hippocampus_L | Frontal_Mid_R |
| 107 | 113 | Frontal_Mid_L | Supp_Motor_Area_L |
| 107 | 114 | Frontal_Mid_L | Supp_Motor_Area_L |
| 107 | 223 | Frontal_Mid_L | Precentral_L |
| 107 | 230 | Frontal_Mid_L | Supp_Motor_Area_L |
| 107 | 254 | Frontal_Mid_L | Supp_Motor_Area_L |
| 107 | 370 | Frontal_Mid_L | Supp_Motor_Area_R |
| 125 | 346 | Precuneus_L | Hippocampus_R |
| 129 | 144 | Frontal_Sup_Medial_L | Frontal_Sup_L |
| 129 | 163 | Frontal_Sup_Medial_L | Calcarine_L |
| 129 | 254 | Frontal_Sup_Medial_L | Supp_Motor_Area_L |
| 129 | 255 | Frontal_Sup_Medial_L | Lingual_L |
| 129 | 363 | Frontal_Sup_Medial_L | Frontal_Mid_R |
| 129 | 400 | Frontal_Sup_Medial_L | Frontal_Sup_R |
| 129 | 419 | Frontal_Sup_Medial_L | Calcarine_R |
| 129 | 485 | Frontal_Sup_Medial_L | Lingual_R |
| 144 | 163 | Frontal_Sup_L | Calcarine_L |
| Source | Target | Source Label | Target Label |
| 144 | 229 | Frontal_Sup_L | Lingual_L |
| 144 | 230 | Frontal_Sup_L | Supp_Motor_Area_L |
| 144 | 254 | Frontal_Sup_L | Supp_Motor_Area_L |
| 144 | 283 | Frontal_Sup_L | Occipital_Mid_R |
| 144 | 465 | Frontal_Sup_L | Temporal_Mid_R |
| 144 | 485 | Frontal_Sup_L | Lingual_R |
| 149 | 346 | Temporal_Sup_L | Hippocampus_R |
| 158 | 192 | Precuneus_L | Thalamus_L |
| 158 | 232 | Precuneus_L | Hippocampus_L |
| 158 | 295 | Precuneus_L | Cingulum_Mid_R |
| 158 | 488 | Precuneus_L | Calcarine_R |
| 159 | 163 | Cingulum_Ant_L | Calcarine_L |
| 163 | 230 | Calcarine_L | Supp_Motor_Area_L |
| 163 | 346 | Calcarine_L | Hippocampus_R |
| 176 | 229 | Cingulum_Mid_L | Lingual_L |
| 184 | 254 | Frontal_Mid_L | Supp_Motor_Area_L |
| 187 | 197 | Frontal_Mid_L | Frontal_Sup_L |
| 189 | 229 | Frontal_Mid_L | Lingual_L |
| 197 | 229 | Frontal_Sup_L | Lingual_L |
| 197 | 230 | Frontal_Sup_L | Supp_Motor_Area_L |
| 197 | 362 | Frontal_Sup_L | Putamen_R |
| 197 | 412 | Frontal_Sup_L | Lingual_R |
| 197 | 452 | Frontal_Sup_L | Putamen_R |
| 197 | 485 | Frontal_Sup_L | Lingual_R |
| 217 | 254 | Temporal_Sup_L | Supp_Motor_Area_L |
| 217 | 370 | Temporal_Sup_L | Supp_Motor_Area_R |
| 224 | 370 | Frontal_Mid_L | Supp_Motor_Area_R |
| 228 | 482 | Frontal_Inf_Oper_L | Frontal_Mid_R |
| 229 | 400 | Lingual_L | Frontal_Sup_R |
| 229 | 447 | Lingual_L | Temporal_Pole_Sup_R |
| 229 | 473 | Lingual_L | Temporal_Sup_R |
| 229 | 486 | Lingual_L | Supp_Motor_Area_R |
| 229 | 506 | Lingual_L | Fusiform_R |
| 230 | 363 | Supp_Motor_Area_L | Frontal_Mid_R |
| 230 | 385 | Supp_Motor_Area_L | Frontal_Sup_Medial_R |
| 230 | 400 | Supp_Motor_Area_L | Frontal_Sup_R |
| 230 | 440 | Supp_Motor_Area_L | Frontal_Mid_R |
| 231 | 254 | Frontal_Sup_Medial_L | Supp_Motor_Area_L |
| 241 | 427 | Temporal_Mid_L | Frontal_Mid_R |
| 254 | 255 | Supp_Motor_Area_L | Lingual_L |
| 258 | 443 | Postcentral_R | Frontal_Mid_R |
| 283 | 321 | Occipital_Mid_R | Occipital_Sup_R |
| Source | Target | Source Label | Target Label |
| 284 | 443 | Frontal_Sup_R | Frontal_Mid_R |
| 284 | 459 | Frontal_Sup_R | Precentral_R |
| 284 | 486 | Frontal_Sup_R | Supp_Motor_Area_R |
| 287 | 459 | Frontal_Sup_R | Precentral_R |
| 311 | 363 | Frontal_Mid_R | Frontal_Mid_R |
| 311 | 400 | Frontal_Mid_R | Frontal_Sup_R |
| 318 | 363 | Frontal_Mid_R | Frontal_Mid_R |
| 355 | 363 | Frontal_Mid_R | Frontal_Mid_R |
| 360 | 440 | Supp_Motor_Area_R | Frontal_Mid_R |
| 363 | 385 | Frontal_Mid_R | Frontal_Sup_Medial_R |
| 363 | 440 | Frontal_Mid_R | Frontal_Mid_R |
| 363 | 482 | Frontal_Mid_R | Frontal_Mid_R |
| 363 | 486 | Frontal_Mid_R | Supp_Motor_Area_R |
| 363 | 510 | Frontal_Mid_R | Frontal_Sup_R |
| 370 | 440 | Supp_Motor_Area_R | Frontal_Mid_R |
| 385 | 400 | Frontal_Sup_Medial_R | Frontal_Sup_R |
| 385 | 510 | Frontal_Sup_Medial_R | Frontal_Sup_R |
| 400 | 486 | Frontal_Sup_R | Supp_Motor_Area_R |
| 400 | 510 | Frontal_Sup_R | Frontal_Sup_R |
| 427 | 460 | Frontal_Mid_R | Frontal_Mid_R |
| 427 | 488 | Frontal_Mid_R | Calcarine_R |
| 440 | 510 | Frontal_Mid_R | Frontal_Sup_R |
| 443 | 451 | Frontal_Mid_R | Precentral_R |
| 482 | 486 | Frontal_Mid_R | Supp_Motor_Area_R |
